# The loss of microRNA-26b promotes aortic calcification through the regulation of cell-specific target genes

**DOI:** 10.1093/cvr/cvaf117

**Published:** 2025-07-30

**Authors:** Diana Luna Buitrago, Eva Jover, Eleonora Mameli, David Mellis, Ryszard Nosalski, Laura Charlton, Rosa Bauza Sanso, Hywel Dunn-Davies, Mark G MacAskill, Adriana A S Tavares, Alexander J Fletcher, Jennifer Nash, Natalia López-Andrés, Marc R Dweck, Patrick W F Hadoke, Derek S Gilchrist, Brad D Wagner, Michael L Robinson, Andrew H Baker, David E Newby, Tijana Mitić, Andrea Caporali

**Affiliations:** University/BHF Centre for Cardiovascular Science, The Queen’s Medical Research Institute, The University of Edinburgh, 47 Little France Crescent, Edinburgh EH16 4TJ, UK; Cardiovascular Translational Research, Navarrabiomed (Fundación Miguel Servet), Instituto de Investigación Sanitaria de Navarra (IdiSNA), Hospital Universitario de Navarra (HUN), Universidad Pública de Navarra (UPNA), Pamplona, Spain; University/BHF Centre for Cardiovascular Science, The Queen’s Medical Research Institute, The University of Edinburgh, 47 Little France Crescent, Edinburgh EH16 4TJ, UK; University/BHF Centre for Cardiovascular Science, The Queen’s Medical Research Institute, The University of Edinburgh, 47 Little France Crescent, Edinburgh EH16 4TJ, UK; University/BHF Centre for Cardiovascular Science, The Queen’s Medical Research Institute, The University of Edinburgh, 47 Little France Crescent, Edinburgh EH16 4TJ, UK; School of Engineering, King’s Buildings, The University of Edinburgh, Edinburgh, UK; University/BHF Centre for Cardiovascular Science, The Queen’s Medical Research Institute, The University of Edinburgh, 47 Little France Crescent, Edinburgh EH16 4TJ, UK; MRC Human Genetics Unit, MRC Institute of Genetics and Cancer, The University of Edinburgh, Edinburgh, UK; University/BHF Centre for Cardiovascular Science, The Queen’s Medical Research Institute, The University of Edinburgh, 47 Little France Crescent, Edinburgh EH16 4TJ, UK; University/BHF Centre for Cardiovascular Science, The Queen’s Medical Research Institute, The University of Edinburgh, 47 Little France Crescent, Edinburgh EH16 4TJ, UK; University/BHF Centre for Cardiovascular Science, The Queen’s Medical Research Institute, The University of Edinburgh, 47 Little France Crescent, Edinburgh EH16 4TJ, UK; School of Cardiovascular and Metabolic Health, BHF Centre of Research Excellence, The University of Glasgow, Glasgow, UK; University/BHF Centre for Cardiovascular Science, The Queen’s Medical Research Institute, The University of Edinburgh, 47 Little France Crescent, Edinburgh EH16 4TJ, UK; School of Cardiovascular and Metabolic Health, BHF Centre of Research Excellence, The University of Glasgow, Glasgow, UK; Cardiovascular Translational Research, Navarrabiomed (Fundación Miguel Servet), Instituto de Investigación Sanitaria de Navarra (IdiSNA), Hospital Universitario de Navarra (HUN), Universidad Pública de Navarra (UPNA), Pamplona, Spain; University/BHF Centre for Cardiovascular Science, The Queen’s Medical Research Institute, The University of Edinburgh, 47 Little France Crescent, Edinburgh EH16 4TJ, UK; University/BHF Centre for Cardiovascular Science, The Queen’s Medical Research Institute, The University of Edinburgh, 47 Little France Crescent, Edinburgh EH16 4TJ, UK; School of Infection and Immunity, College of Medical Veterinary and Life Sciences, The University of Glasgow, Glasgow, UK; Department of Biology and Centre for Visual Sciences, Miami University, Oxford, OH, USA; Department of Biology and Centre for Visual Sciences, Miami University, Oxford, OH, USA; University/BHF Centre for Cardiovascular Science, The Queen’s Medical Research Institute, The University of Edinburgh, 47 Little France Crescent, Edinburgh EH16 4TJ, UK; Department of Pathology, CARIM, Maastricht University, Maastricht, The Netherlands; University/BHF Centre for Cardiovascular Science, The Queen’s Medical Research Institute, The University of Edinburgh, 47 Little France Crescent, Edinburgh EH16 4TJ, UK; University/BHF Centre for Cardiovascular Science, The Queen’s Medical Research Institute, The University of Edinburgh, 47 Little France Crescent, Edinburgh EH16 4TJ, UK; University/BHF Centre for Cardiovascular Science, The Queen’s Medical Research Institute, The University of Edinburgh, 47 Little France Crescent, Edinburgh EH16 4TJ, UK

**Keywords:** single-cell transcriptomics, microRNAs, aortic calcification, cell–cell communications, aortic disease

## Abstract

**Aims:**

Vascular calcification is the abnormal deposition of calcium phosphates within blood vessels. This condition is significantly associated with the development of cardiovascular disease, yet the underlying mechanisms remain largely unknown. MicroRNAs (miRNAs) may be crucial in initiating vascular calcification by regulating a network of specific cellular targets. In this study, we explored for the first time the potential role of microRNA-26b (miR-26b) in vascular calcification.

**Methods and results:**

Using micro-positron emission tomography and computed tomography (micro-PET/CT) imaging with ^18^F–sodium fluoride, we measured aortic calcification in miR-26b knockout mice (miR-26bKO). We conducted bulk RNA sequencing (RNA-seq), single-cell RNA sequencing, and network analysis to identify cell-specific targets and the cellular complexity contributing to the observed phenotype. Additionally, we examined aortic tissues from patients with aortic aneurysm or valvular-related aortopathy to determine how the expression levels of miR-26b and its targets correlate with calcification. Our findings revealed that miR-26b is downregulated in the aortic tissues of patients with aortic calcification, whereas miR-26b expression negatively correlates with calcification levels. Similarly, miR-26bKO mice developed spontaneous age-related aortic microcalcifications. Combining single-cell transcriptomics with network analyses, we identified and mapped cell-type specific targets of miR-26b and regulatory pathways. Furthermore, we validated the cell-specific expression of *Smad1* in smooth muscle cells (SMCs) and characterized the cell–cell communication between aortic cells, exposing the bone morphogenetic protein (BMP) pathway. The development of microcalcification was attributed to Bmp4 released from fibroblasts (FBLs), leading to Smad1 phosphorylation and calcium accumulation in SMCs of miR-26bKO mice. We found that aortic microcalcification could be pharmacologically reversed by disrupting cellular communication. Lastly, we demonstrated an inverse correlation between miR-26b and *SMAD1* levels in calcified aortic tissues.

**Conclusion:**

The deficiency of miR-26b is crucial for initiating and promoting aortic calcification, revealing new therapeutic targets for aortic disease.

## Graphical Abstract

**Graphical Abstract:**
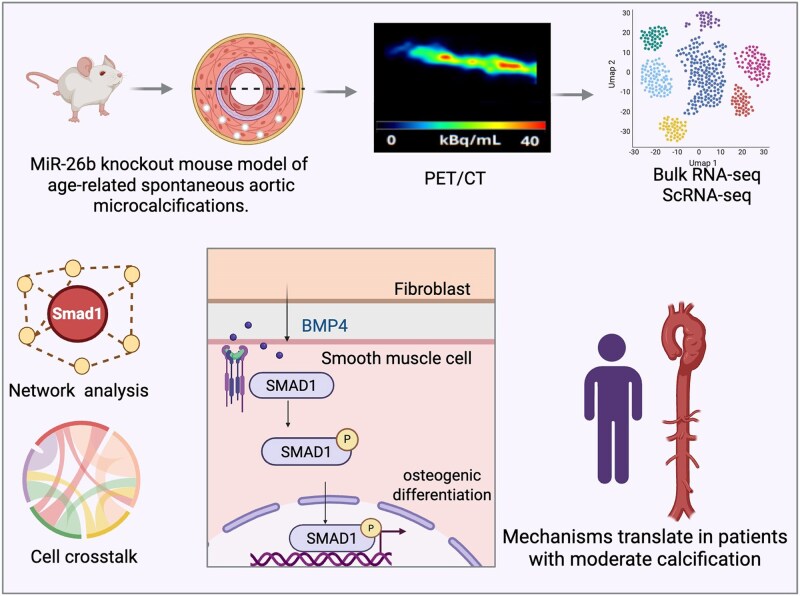

**Time of primary review: 27 days**

## 1. Introduction

Vascular calcification (VC) results from a pathological deposition of calcium phosphate minerals in soft tissues.^1^ Symptoms and associated risks vary depending on the site (e.g. artery vs. valve, tunica media vs. intima) and the nature of calcification (micro- vs. macro-calcification).^2^ While advanced age and male sex constitute risk factors for VC, this condition often arises as a complication of disease states such as diabetes mellitus, chronic kidney disease, atherosclerosis, hypertension, or dyslipidaemia.^3^ Clinical consequences may include aortic stiffening or aortic valve stenosis, ultimately leading to the malfunction of affected organs. Accordingly, VC in the arterial wall is associated with an increase (over 4-fold) in the risk of all-cause mortality, and it is also a strong predictor of cardiovascular morbidity, including hypertension, heart failure, and myocardial and limb ischaemia.^4^ Microcalcification generally reflects the earliest and most active stages of mineralization (propagation phase)^5^ and can be detected using ^18^F–sodium fluoride (^18^F–NaF), which binds specifically to hydroxyapatite (calcium phosphate crystals).^6,7^ Recent studies showed that the degree of microcalcification, as detected by ^18^F-NaF positron emission tomography (PET), has predicted clinical events in the abdominal aortic aneurysms^8^ and was positively associated with the mild and moderate histopathological changes in thoracic aortic aneurysm.^9^

VC is a cell-mediated process, and the acquisition of an osteoblast-like phenotype by healthy vascular cells in response to pro-calcific signals is central to the pathogenesis of this disease.^10^ However, the mechanisms driving the early aspects of this vascular-to-osteoblast-like cell transition still need to be fully understood. They are likely dependent on the cell type, the vascular bed, the VC type, and the presence or absence of underlying diseases. In arteries, the early stages of medial calcification are driven by a breakdown of the extracellular matrix and phenotypic change of the vascular smooth muscle cells (SMCs).^10^ In addition, SMCs can undergo a phenotypic switch to an osteo-chondrogenic phenotype when calcification inhibitors are lost, or in response to hyperphosphataemia, reactive oxygen species (ROS), or pro-inflammatory mediators.^11^ Activated adventitial fibroblasts (FBLs) can contribute to the progression of vascular calcification, and potential mechanisms might be associated with FBL-secreted pro-inflammatory cytokines and growth factors.^12^ Accumulating evidence describes the initiation of calcification as a tightly regulated process^13^; however, the precise molecular and cellular mechanisms initiating this process are still unclear.

MicroRNAs (miRNAs) are single-stranded non-coding RNAs comprising ∼22 nucleotides. They act as post-transcriptional repressors by targeting the 3′-untranslated region of messenger RNAs (mRNAs) in >60% of human genes.^14^ Importantly, miRNAs regulate gene expression (GEX) in a cell-specific manner,^15^ enabling their target genes to shape distinct phenotypes and responses to stimuli. Emerging studies reported that miRNAs function as regulators of cardiovascular calcification^16^ and SMC trans-differentiation into an osteogenic phenotype.^17^

The miR-26 family has recently been associated with cardiovascular disease and pathological angiogenesis.^18^ The human and mouse miR-26 family consists of miR-26a-1, miR-26a-2, and miR-26b, localized on three different chromosomes. The mature miR-26a-1 and miR-26a-2 have identical sequences, differing from the mature miR-26b sequence by just two nucleotides, affecting target gene regulation.^18^ We previously described the role of miR-26b in the vasculature, showing that it can protect endothelial cells (ECs) and myocytes from apoptosis during ischaemic injury.^19^ Our bioinformatics analysis of putative miR-26b target genes identified a significant enrichment of targets belonging to the BMP/TGFβ pathway,^19^ suggesting a potential role in promoting VC. Consequently, we designed experiments to investigate how miR-26b cell-specific target genes and vascular cell–cell communication could initiate and promote vascular calcification.

## 2. Methods

Detailed procedures are described in the Supplementary Materials.

### 2.1 Human samples

This study complies with the ethical principles recorded in the 1975 Declaration of Helsinki and later amendments. Specimens of ascending aortic tissues were obtained from patients diagnosed with severe aortic valve disease (e.g. aortic regurgitation or aortic stenosis) and aortopathy elective of concomitant surgical aortic valve and aorta replacement (e.g. Bentall procedure) as well as patients with lone aortopathy elective for valve-sparing aortic root replacement (e.g. David procedure) at Hospital Universitario de Navarra, Spain. The study was covered by the Research Ethics Committee approval (Pyto. 2013/26, num 137) in agreement with the Spanish law (BPCCPMP/ICH/135/95). The clinical and demographic characteristics of the enrolled patients are described in Supplementary material online, *Table S1*. Specimens of aneurysmal thoracic aortic tissue were obtained from the Royal Infirmary of Edinburgh, United Kingdom. Samples were collected from consenting patients or relatives under the ethical approval (15/ES/0094). The clinical and demographic characteristics of the enrolled patients were previously described in Ref.^9^.

### 2.2 Murine models and *in vivo* experiments

All experiments involving mice were performed following guidance and operation of the Animals (Scientific Procedures) Act 1986 (2014) with prior approval of the UK Home Office (PP3528002) and the University of Edinburgh Animal Welfare and Ethical Review Board. The results were reported following the ARRIVE guidelines.

The miRNA-26b global knock-out (KO) mouse was generated using CRISPR/Cas9 via FVB/N zygote microinjection^20^ and provided by Michael L Robinson’s lab (Department of Biology and Centre for Visual Sciences, Miami University, Oxford, OH, USA) (primers and gRNA sequences listed in Supplementary material online, *Table S2*). miRNA-26bKO mice were backcrossed to the C57BL/6J background and housed, bred and maintained at the University of Edinburgh Little France Facility. All animals were kept in a dedicated pathogen-free animal facility with 12-h light/12-h dark cycles and *ad libitum* access to food and water. Wild type (WT) and miR-26bKO mice received either LDN-193189 (i.p. 3 mg/kg/day) or vehicle (DMSO) every second day for 14 days (*n* = 8/group). Mice were sacrificed by asphyxiation with rising concentration of carbon dioxide gas, followed by cervical dislocation and tissue collection. The endpoints of this study measured the levels of calcification with histological analysis. For *in vivo* studies, the sample size for each experiment is indicated in the figure legend. This study analysed male and female mice, with the number and sex of the mice specified in the legends for each experiment.

### 2.3 Aortic cell isolation

Primary fibroblasts (FBLs) and vascular smooth muscle (SMCs) cells were obtained from 8 to 10 weeks old WT or miR-26bKO mice. Aortas were cleaned from periaortic fat, adventitia was separated mechanically from media, and the obtained layers were placed separately into an enzyme solution (Collagenase II, Elastase, Soybean Trypsin Inhibitor in HBSS with calcium and magnesium) for 1 h at 37°C in 5% CO_2_ in the incubator. Cells were cultured in DMEM (Gibco) supplemented with 10% FBS until 90% confluent. Cells were cultured in DMEM/F12 (Gibco), supplemented with 20% FBS until 90% confluent. Endothelial cell suspensions were stained for CD31 and CD45 antibodies, washed and re-suspended in PBS at 4°C and analysed using a BD 5L LSR Fortessa and BD FACSDiva software (BD Biosciences). Cells were sorted using a FACS Aria II instrument and BD FACSDiva software (BD Biosciences).

### 2.4 RNA isolation, RT, and qPCR

Total RNA was isolated using Qiazol reagent and miRNeasy miniKit (217004, QIAGEN), and reverse-transcribed into single-stranded cDNA, using an iScript Advanced cDNA Synthesis Kit (Bio-Rad) for mRNA analyses. Downstream qPCR amplification was performed using iQ SYBR Green Supermix (Bio-Rad) in a CFX Connect Real-Time PCR System (Bio-Rad) using primers at 300 mM final concentration. The validated primers are commercially available from Sigma (KICqStartTM Primers). *GAPDH*, *ACTA2*, *18S*, and *HRPT* were used as housekeeping genes. To study the expression of miR-26b-5p, specific Taqman microRNA assay primers and probes were used (TaqMan™ microRNA Control Assay U6 snRNA, Cat #4427975, assay ID:001973 and Taqman™ probe miR-26b-5p, Cat #4427975, assay ID:000407, respectively). Reverse transcription was performed with the TaqMan™ MicroRNA Reverse Transcription Kit (Applied Biosystems) using 10 ng total RNA. Downstream qPCR amplifications of first-strand cDNA were performed using TaqMan® Universal PCR Master Mix, no AmpErase® UNG(4324018). U6 snRNA was used as a housekeeping gene for miRNA determination. The relative expression of each selected gene product was calculated using the 2^−ΔΔCt^ method.^21^ All reactions were performed in technical triplicates.

### 2.5 *Ex vivo*  ^18^F-NaF micro-PET and computed tomography scans

The whole mouse aorta was harvested from 3- and 6-month-old wild-type and miR-26bKO male and female mice. The aorta was dissected from surrounding tissue, and the perivascular fat was removed. Aortas were incubated with 103–127 kBq/mL ^18^F-NaF in PBS for 30 min and washed twice in PBS. The tissue was then placed in the scanner, and a 30-min PET scan was performed using a three-dimensional 1:5 mode. A CT scan (semi-circular full trajectory, maximum field of view, 720 projections, 50 kVp, 300 ms, and 1:4 binning) was acquired for attenuation correction and quantification of microcalcification. Scans were reconstructed using Mediso’s iterative Tera-Tomo 3D reconstruction algorithm. PET data were analysed using PMOD version 4.2 (PMOD Technologies, Switzerland). Briefly, volumes of interest were drawn around the aortic arch and thoracic aorta. The mean signal across the 10 highest-intensity voxels within that volume of interest was then calculated using the hot average function and normalized to the target concentration of 100 kBq/mL. PET images were created by scaling the images using the radiotracer incubation concentration relative to the target concentration and applying a 0.5 × 0.5 × 0.5 Gaussian filter.

### 2.6 Immunohistochemistry and miR-26b *in situ* hybridization

DAB staining for SMAD1 (pSMAD1/5, Invitrogen #700047) was performed using the Leica Bond III autostainer (Leica Biosystems, USA) and Bond Polymer Refine Detection kit (DS9800, Leica Biosystems, USA). *For in situ* hybridization: Slides were then hybridized overnight using a hybridization solution (K2191050, Amsbio) at 45°C using 80 nM detection digoxigenin probes (miRCURY LNA miRNA Detection probe, Qiagen). After blocking, slides were incubated in alkaline phosphatase-conjugated anti-digoxigenin antibody (1:100 dilution) (K2191050, Amsbio, UK) for 4 h at 4°C. Tissues were then imaged using Axio Scan Z.1 (Zeiss, Oberkochen). Representative negative control images consisting of IgG isotype controls are displayed in Supplementary material online, *Figure S7B*–*D*. Image analysis pipelines are reported in Supplementary Methods.

### 2.7 Western blot analysis

Total proteins were extracted in RIPA buffer containing 1 mM sodium orthovanadate and Complete Protease Inhibitor Cocktail (Roche Applied Science) and quantified using the Pierce™ BCA protein assay kit (Thermo Fisher). Equal amounts of proteins were loaded onto SDS–polyacrylamide gels and transferred to nitrocellulose membrane. The membranes were then blocked with Intercept (TBS) Blocking Buffer with Tween 20 at 0.2% and immunoblotted overnight at 4°C with the following primary antibodies (1:1000): phospho-SMAD1 (Ser463/465) (41D10) (CST, #9516), total SMAD1 (Proteintech, 66559-1-Ig), and β-actin (Sigma, A5441). Secondary antibodies (1:15,000): IRDye® 680RD anti-Mouse IgG (926-68070) and IRDye® 800CW anti-Rabbit IgG (926-3221) were incubated for 1 h at RT. Membranes were scanned using Odyssey F instrument (LICORbio). Analysis and quantification were performed using Empiria Studio® Software (LICORbio).Uncropped blots are provided in Supplementary material online, *Figure S7E*.

### 2.8 Bulk RNA-sequencing and bioinformatics analysis

Bulk RNA-seq to determine the transcriptomes of the aorta of 6-month-old male mice (wild-type *n =* 3 and miR-26bKO *n =* 3) was performed by Beijing Genomics Institute (BGI) Company (Shenzhen, China). The sequencing was performed using DNBSEQ system. Bioinformatics analysis was conducted using Dr Tom (BGI online platform). Only genes with transcripts per million (TPM) > 1 were analysed. Differentially expressed genes (DEGs) were identified using the DEGseq2 method and selected using *P*_adj_ value ≤ 0.05 and log2FC ≥ 1.

### 2.9 Single-cell RNA-sequencing

Single-cell preparation: The single-cell suspension of aortic cells was performed based on a published enzymatic digestion protocol.^22^ Three aortas from male mice were pooled per sample, and after FACS sorting, *n* = 3 samples per group (WT vs. KO) were analysed.

Library preparation and sequencing: Cells were prepared using 10× Genomics Chromium Next GEM Single Cell 3′ Reagent Kits v3.1 with Feature Barcode technology for cell multiplexing (CG000389) following manufacturer instructions. After resuspension and before incubation with antibodies for FACS sorting, cells were stained using 10× Genomics CellPlex CMO oligos to hash individual samples (see Supplementary material online, *Table S3*). Each cell pool was resuspended in an appropriate volume of PBS + 1%BSA, and a maximum of 60 000 cells per pool were loaded on a 10× Genomics Chip G with V3.1 gel beads and partitioning oil. Cells were then encapsulated with gel beads using a 10× Genomics Chromium X instrument. After encapsulation and cDNA synthesis, each reaction was amplified by PCR, and both low- and high-MW fractions were purified separately. The high-MW fraction containing cDNA was fragmented, ligated with sequencing adapters and amplified to generate GEX libraries. The low-MW fraction was further amplified with sequencing adapters to generate multiplexing (MP) libraries. All libraries were pooled at a molar ratio of GEX:MP = 10:1 and sequenced on an Illumina NextSeq 2000 P3 flow cell with 100 cycles configuration.

Single-cell RNA-seq data analysis was performed using the cellranger multi command from 10× Genomics Cell Ranger v7.1.0 (MAC value of 0.5 to provide demultiplexed cell-specific GEX data). As an alternative approach, the nf-core single-cell RNA-sequencing (scRNA-seq) pipeline (doi:10.5281/zenodo.3568187) v2.3.0 was also run on FASTQ files using both Salmon v1.10.0 paired with Simpleaf v0.10.0,^23^ and cellranger count (mkfastq) from 10× Genomics Cell Ranger v7.1.0. The scRNA-seq dataset (*n* = 5191 cells) was then processed, explored, and visualized using Trailmaker (Parse Biosciences; https://app.trailmaker.parsebiosciences.com/). The Data Processing settings are in Supplementary material online, *Table S4*. Analysis of cell–cell communication networks was performed in ICARUS (Interactive Single Cell RNA-seq Analysis with R shiny Using Seurat)^24^ web server using the CellChat R package.^25^

### 2.10 Network analysis and miRNA target predictions

Cytoscape (Version 3.9.1) is a freely available software tool which facilitates the creation of networks, allowing for the visualization of molecular interactions (https://cytoscape.org/).^26^ Additional features are present in Cytoscape, in the form of Apps. StringApp (Version 2.0.3) was utilized to import established gene-to-gene interactions through the protein query. DIANA-miRPath v4.0^27^ and TargetScan 8.0^28^ were utilized for miR-26b target gene prediction and enrichment.

### 2.11 Statistical analysis

Normal data distribution was assessed using Kolmogorov–Smirnov or Shapiro–Wilk’s tests as appropriate. Continuous variables are shown as the mean ± standard error of the mean (SEM). Categorical variables are presented as counts (percentages). Normally distributed variables were analysed using the unpaired two-tailed Student’s *t*-test (two-group comparisons) or one-way analysis of variance (multiple-group comparisons, ANOVA), as appropriate. ANOVA post hoc analysis was performed using the Bonferroni approach as appropriate. Data with two factors were analysed by two-way ANOVA followed by Tukey *post hoc* analysis. Non-parametric tests, including the Wilcoxon/Mann–Whitney *U* test or the Kruskal–Wallis test, were used for data that was not normally distributed. Pearson or Spearman linear regression was calculated to study the relationship among the continuous variables of interest. A *P-*value <0.05 was considered statistically significant. Analyses were performed using GraphPad Prism v5.0.

## 3. Results

### 3.1 miR-26b is downregulated in the aortic tissues of patients with aortic calcification

**Figure 1:**
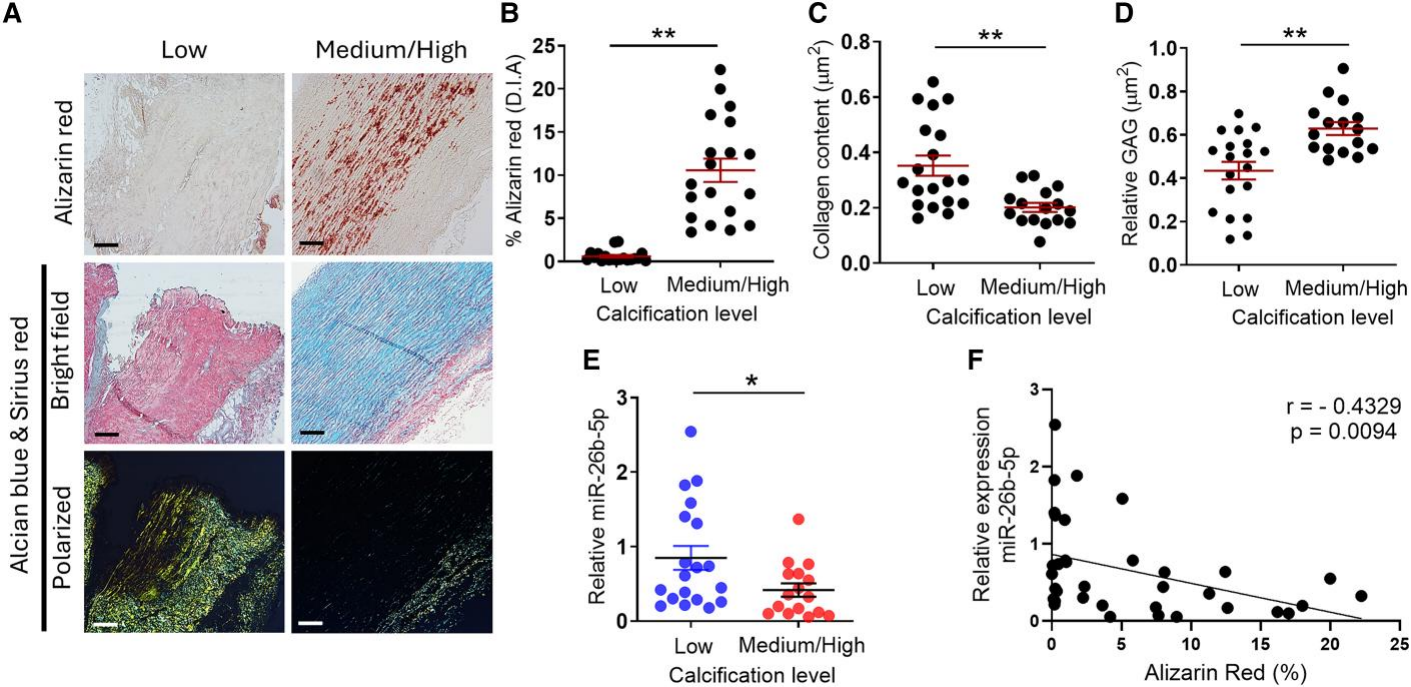
miR-26b-5p is downregulated in the aortic tissues of patients with aortopathy and aortic valve disease. (*A*) Histological characterization of human aortas (aortopathy concomitant or not with aortic valve disease, see Supplementary material online, *Table S1*) was performed by alizarin red staining (top panel), Sirius red/alcian blue double staining in bright field (middle panel) and polarized light (bottom panel). Scale bar = 200 μm. (*B*) Calcification was quantified as % of alizarin red staining, with a significant increase of calcium content within the ‘medium/high’ vs. the ‘low’ group. Extensive ECM was evidenced using Sirius red/alcian blue double staining. (*C*) Collagen and (*D*) GAG accumulation were quantified in different calcification groups. (*E*) The expression of miR-26b-5p in low, medium/high calcification samples and (*F*) correlation with the content of calcium (% alizarin red) (*r* = −0.4329, *P* = 0.0094; Spearman correlation; *n* = 35). For (*B*–*E*): *n* = 19 low (5 female/14 male) and *n* = 16 medium/high (4 female/12 male); *P < 0.05; **P < 0.01 vs. low calcification; Student’s unpaired *t*-test. All data are mean ± SEM.

We analysed aortic tissues from patients with concomitant aortopathy and aortic valve disease to investigate whether miR-26b-5p is downregulated during vascular calcification (clinical features are shown in Supplementary material online, *Table S1*). Aortic tissues were sub-grouped according to low (<2%) and medium/high calcification (>2%) by Alizarin red stain digital image analysis^29^ (*Figure 1A* and *B*). Aortic tissues were also co-stained with Alcian blue and Sirius red to reveal glycosaminoglycan (GAG) and collagen composition, respectively (*Figure 1A*). Image analysis revealed a significant extracellular matrix remodelling (ECM) within human aortic tissues, showing a decrease in collagen deposition (*Figure 1C*) and an increase in GAG accumulation (*Figure 1D*) in correlation with a higher level of calcification. Consequently, the collagen content was inversely correlated with calcium levels (*r* = −0.4431, *P* = 0.0098), while GAG accumulation displayed a positive correlation (*r* = 0.3445, *P* = 0.0496), as shown in Supplementary material online, *Figure S1A* and *B*. However, we did not observe a significant correlation between the level of aortic miR-26b expression and collagen or GAG deposition (see Supplementary material online, *Figure S1C* and *D*). MiR-26b-5p expression was significantly decreased in aortic tissues with medium/high calcification compared with the low calcification group (*Figure 1E*), and its expression was negatively correlated with calcification levels (*Figure 1F*).

### 3.2 miR-26bKO mice develop spontaneous aortic microcalcifications

To investigate the cardiovascular phenotype of miR-26b, we generated a miR-26bKO mouse using CRISPR/Cas9 technology.^20^ DNA sequencing analysis demonstrated the deletions in the miR-26b sequences with the partial loss of the miR-26b seed sequence (red letters) in the KO mice (see Supplementary material online, *Figure S2A*). The expression of mature miR-26b-5p and miR-26b-3p was abolished entirely in the miR-26bKO aortas compared with wild-type (WT) mice; however, the deletion of miR-26b did not affect the expression of miR-26a, miR-26a-2 or *Ctdsp1*, the miR-26b host gene (see Supplementary material online, *Figure S2A*).

**Figure 2:**
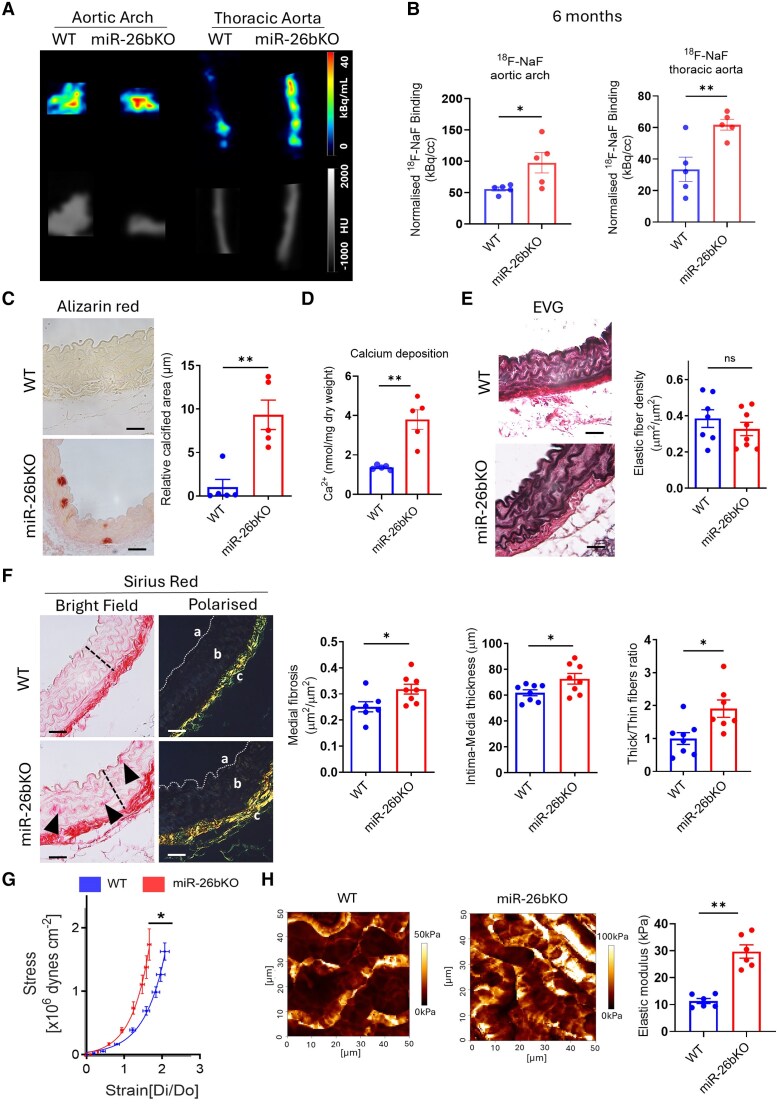
miR-26bKO mice develop spontaneous aortic microcalcifications. (*A*) Representative *ex vivo* micro-PET/CT scans of the aortic arch and thoracic aorta in WT and miR-26bKO mice at 6 months. The colours correspond to the strength of the ^18^F-NaF (hydroxyapatite marker) signal. (*B*) Quantification of microcalcification in the aortic arch and thoracic aorta (*n* = 5 per group). (*C*) Representative images of alizarin red staining from WT and miR-26bKO aortas (left) and relative fold-change of calcified areas (right) (*n* = 5 per group). Scale bar = 50 μm. (*D*) Calcium deposition in the aortas (*n* = 5 per group). (*E*) Representative images of Elastic Van Giesson (EVG) staining from WT and miR-26bKO male aortas (left) and measured elastic fibre density (right), *n* = 7–8 per group. Scale bar = 50 μm. (*F*) Images of Sirius red-stained mouse aortas in brightfield (left) and polarized light (right), along with medial fibrosis, Intima-media thickness and Thick/thin fibres ratio measurement, *n* = 7–8 per group. Scale bar = 50 μm. Black arrows = interstitial fibrosis deposits. Dotted line = medial thickness. Luminal (*A*), medial (*B*), and adventitial (*C*) areas. Dotted lines delimit the luminal area. (*G*) Abdominal aorta stiffness was studied by pressure myography, (*n* = 5–6 per group; male mice). (*H*) Young’s modulus image mappings within mouse aorta (left) and media stiffness measurements measured by atomic force microscopy (right) (*n* = 6 per group). Male mice have been used in these experiments. For (*B–F* and *H*): ns = non-significant; *P < 0.05 vs. WT; **P < 0.01 vs. WT. Student’s unpaired *t*-test. For (*G*): *P < 0.05 vs. WT; two-way ANOVA. All data are mean ± SEM. Low-magnification images of the aortic rings have been displayed in Supplementary material online, Figure  *S3A* and *B*.

We used ^18^F-NaF micro-PET/CT to detect the level of calcification in these mice. Upon dissection of WT and miR-26bKO mice at 3 or 6 months of age, aortas were probed with ^18^F-NaF, a hydroxyapatite probe for subsequent micro-PET/CT scanning to visualize microcalcification. We found no significant difference in the accumulation of ^18^F-NaF between WT and miR-26bKO mice at 3 months of age (see Supplementary material online, *Figure S2B* and *C*). However, at 6 months of age, scans showed a significant increase in ^18^F-NaF detected signal (microcalcification) in the aortic arch and the thoracic aorta of miR-26bKO mice (*Figure 2A* and *B*). CT scan analysis did not reveal any sign of macro-calcification in the aorta of miR-26bKO mice (*Figure 2A*).

Additionally, we examined the presence of microcalcifications in the aortas of 6-month-old female WT and miR-26bKO mice (see Supplementary material online, Figure  *S2D* and *E*). The miR-26bKO female mice exhibited spontaneous development of microcalcifications, like the male KO mice, indicating no sex-dependent effect on the phenotype in this mouse model.

Histological analysis using Alizarin red stain confirmed calcium accumulation in the thoracic aorta of miR-26bKO compared to WT (*Figure 2C* and Supplementary material online, *Figure S3A*). This was further confirmed using an o-cresolphthalein assay (*Figure 2D*). Although microcalcification was present, we did not observe elastin fibre disruption or loss of the elastin network in the medial layer in these samples (*Figure 2E* and Supplementary material online, *Figure S3A*). Moreover, the Sirius red stain showed a significant increase in intima-media thickness, collagen accumulation in the medial layer of the miR-26bKO aorta, and significant changes in fibre thickness in the medial layer by using polarized light (*Figure 2F* and Supplementary material online, *Figure S3A*). In line with these results, pressure myography on mouse aortas showed a leftward shift of the strain/stress curve in miR-26bKO mice compared to WT (*Figure 2G*). Similarly, atomic force microscopy analysis determined a significant increase in Young’s modulus, thus confirming increased aortic stiffness in miR-26bKO (*Figure 2H* and Supplementary material online, *Figure S3B*). Although the presence of aortic stiffness, echocardiography performed in both miR-26bKO and WT at 6 months of age did not show significant changes in parameters of cardiac structure such as left ventricle (LV) dimensions, including LV thickness and LV mass (see Supplementary material online, *Figure S4A* and *B*) or in parameters of cardiac functionality (see Supplementary material online, *Figure S4C*).

### 3.3 Single-cell transcriptomics revealed cellular and molecular changes in miR-26bKO aortas

**Figure 3:**
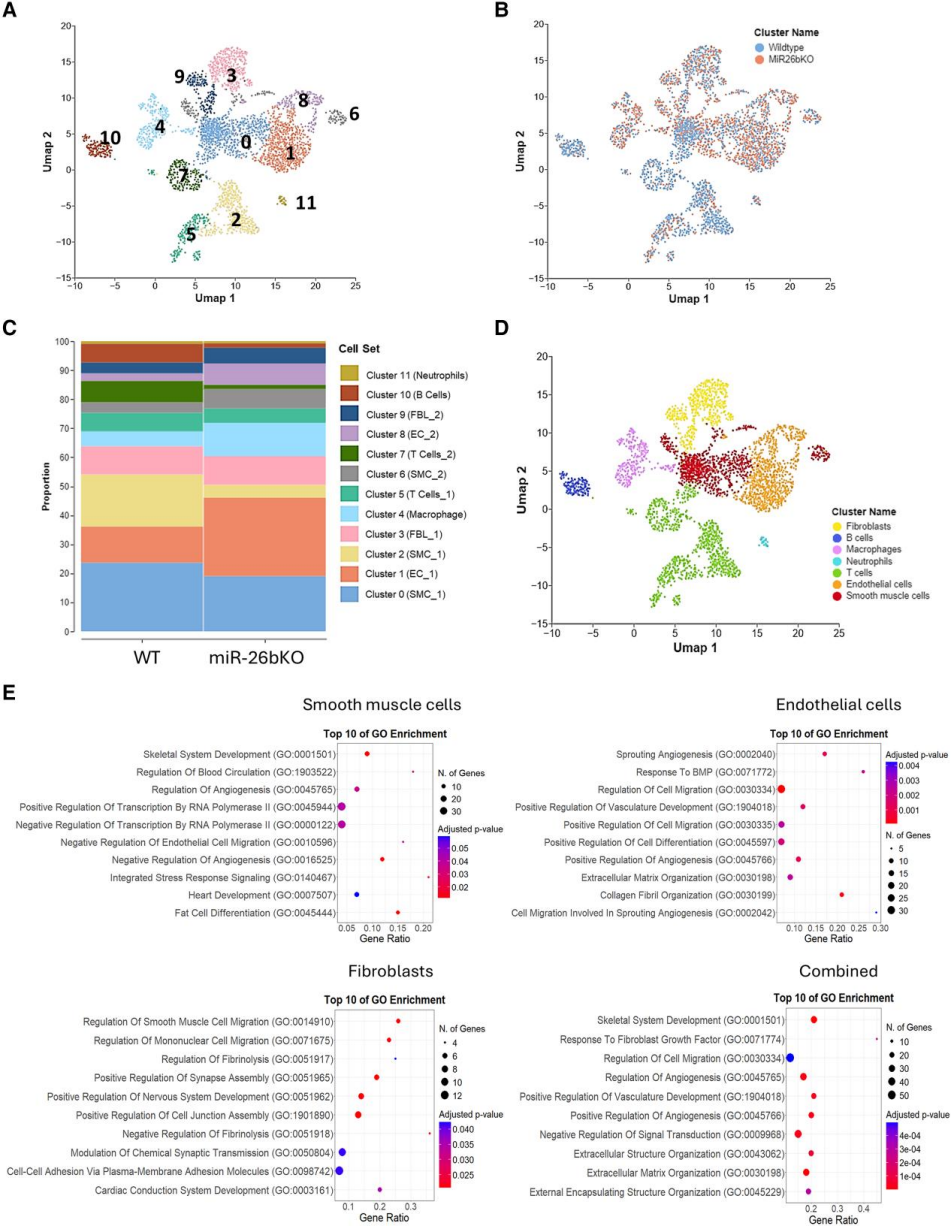
Clustering and mapping of cell groups in miR-26bKO mouse aorta. Single-cell transcriptomic analysis was performed in cells isolated from mouse aortas from 6-month-old WT and KO male mice (*n* = 5191). (*A*) UMAP plot composed of whole aortas from WT and KO mice, showing 12 cell clusters. (*B*) UMAP plot, composed of whole aortas from WT and KO mice, shows the total cell distribution. (*C*) Cluster frequency analysis in the aorta of WT and KO mice. (*D*) UMAP projection of the annotated clusters representing main cell types residing in the aorta. (*E*) Dot plot displaying the gene enrichment for gene ontologies (GO: Biological Process) (log10 of the false discovery rate) for cell types: ECs, fibroblasts (FBLs) and vascular smooth muscle cells (SMCs), comparing GEX levels between WT and miR-26bKO. The gene ratio (*x*-axis) indicates the number of overlapping genes divided by the total number of reference genes in the gene ontology (GO) category.

To map the cellular composition of WT and miR-26KO aortas, single-cell transcriptomic analysis was performed on isolated cells (*n* = 5191) from mouse aortas of 6-month-old WT and miR-26bKO. Cluster analysis using the uniform manifold approximation (UMAP) and projection technique, has identified 12 cell clusters in the aortic tissue. We assigned clusters to their putative identities and hierarchical similarities by differentially expressed gene signatures (*Figure 3A*). The knock-out of miR-26b has produced substantial changes in total cell distribution and cluster frequency (*Figure 3B* and *C*). The cluster-specific marker genes were visualized in dot plots (see Supplementary material online, *Figure S5A*). The resulting 12 clusters were manually annotated to a general cell type level with the following marker genes^30–32^: macrophage (*Csf1r, Cd68, Adgre1*), smooth muscle cells (SMCs) (*Myh11, Tagln, Acta2, Mylk*), ECs (*Pecam1, Cdh5, Cldn5, vWF*), fibroblasts (FBLs) (*Fbln1, Pdgfra, Serpinf1, Col1a1*), T Cells (*Cd2, Cd3d, Cd3e, Lef1*), B Cells (*Cd19, Cd27, Ms4a1, Cd38*), and neutrophils (*S100a8, S100a9, Retlng*) (*Figure 3D*).

MA plots were generated to analyse and interpret DEGs (log_2_FC = 1.5; *P*_adj_ ≤ 0.05) for each cell type (see Supplementary material online, *Figure S5B*). We then conducted pathway analysis using DEGs from the FBL, SMC, and EC populations by comparing genes from miR-26bKO with WT mice (*Figure 3E*). According to gene ontology (GO) analysis (biological process), the dominant biological processes of these DEGs were ‘Regulation of transcription by RNA Polymerase II (negative or positive)’ in SMCs, ‘Regulation of cell junction assembly’ in FBLs, and ‘Regulation of cell migration’ in ECs.

### 3.4 Network analysis identified cell-specific mechanisms and drivers of aortic calcification

**Figure 4:**
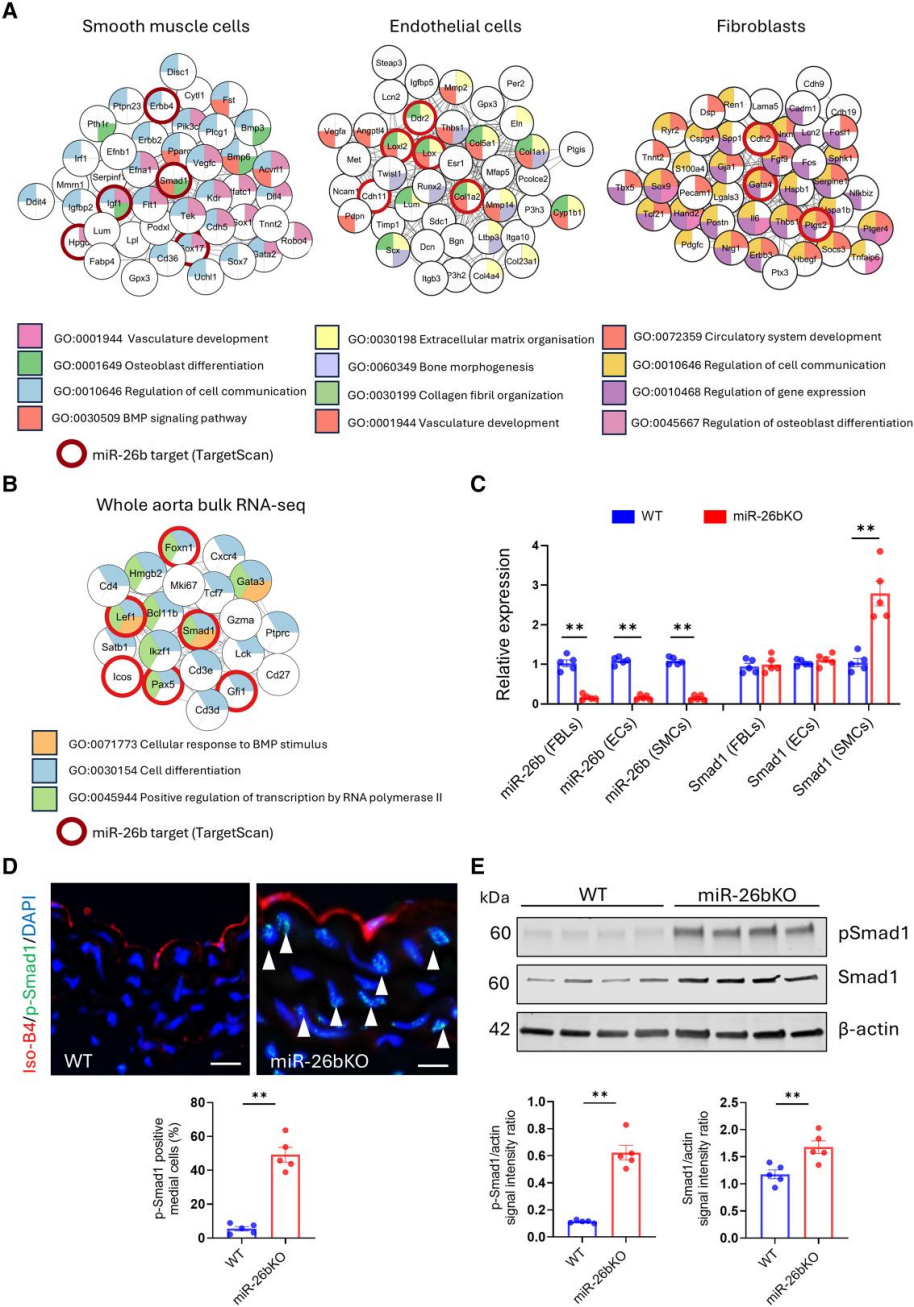
Network analysis of miR-26b target genes in the aorta of miR-26bKO mice. (*A*) Network analysis of DEGs between WT vs. KO mouse in smooth muscle cells (SMCs), ECs and fibroblasts (FBLs) (scRNA-Seq) and (*B*) whole aorta (bulk RNA-Seq). miR-26b targets are circled in red (*P*_adj_ < 0.05). (*C*) Quantification of GEX of miR-26b-5p and *Smad1* within ECs, FBLs and SMCs from WT and miR-26bKO mouse aortas (*n* = 5 per group). (*D*) Representative immunohistochemical images and quantification of p-Smad1 media localization across WT and miR-26bKO mouse aortas samples (*n* = 5 per group). White arrows: p-Smad1 positive cells. Scale bar = 100 μm. (*E*) Representative Western blots for p-Smad1 (Ser463/465), total Smad1 and β-actin in WT and mir-26bKO mouse aortas and quantification of signal intensity compared to β-actin (loading control) (*n* = 5 per group). Male mice have been used in these experiments. For (*C–E*): **P < 0.01 vs. WT; One-way ANOVA. All data are mean ± SEM.

Network-based methods facilitate the integration of transcriptomics to identify and prioritize key molecular interactions or disease drivers.^33^ Moreover, scRNA-seq data can help map the cell context-specificity of miRNA target genes, thus determining cell-specific signalling regulators. Therefore, we performed a network analysis between upregulated genes in SMCs, ECs, and FBLs in the aortas of miR-26bKO mice and predicted target genes from TargetScan^28^ to identify miR-26b-enriched target gene pathways that could determine the phenotype observed in miR-26bKO mice (*Figure 4A*).

The network analysis demonstrated that cell-specific drivers are involved in BMP signalling pathway (GO:0030509) and Osteoblast differentiation (GO:0001649) in SMCs (key node *Smad1*); Extracellular matrix organization (GO:0030198) in ECs (key node *Col1a2*) and regulation of GEX (GO:0050727) in FBLs (key node *Gata4*). Moreover, this approach showed that genes involved in the calcification process were present in each cell type (GO:0030509 BMP signalling pathway and GO:0001649 osteoblast differentiation for SMCs; GO:0060349 bone morphogenesis for ECs; GO:0045667 regulation of osteoblast differentiation for FBLs). We then applied the same approach to bulk RNA-seq of aortas isolated from WT and miR-26bKO. In total of 717 DEGs were obtained (532 upregulated and 132 downregulated; using log2FC ≥ 1, *P*_adj_ ≤ 0.05); also confirming that *Smad1* can be identified as a key node in this model amongst the top upregulated miR-26b target genes (*Figure 4B*).

To validate SMC-specific regulation of *Smad1*, we isolated FBLs, SMCs (enzymatic digestion) and ECs (flow cytometry CD31^pos^ CD45^neg^) from WT and miR-26KO mice (*Figure 4C*). The type of isolated cells was further confirmed by the expression of markers used for the scRNA-seq analysis (see Supplementary material online, *Figure S5A* and *B*). miR-26b-5p was downregulated in FBLs, ECs and SMCs, whereas *Smad1* was upregulated only in SMCs (*Figure 4C*). Immunohistochemistry confirmed the localization of phospho-Smad1(Ser463/465) (p-Smad1) in aortic SMCs of miR-26bKO mice (*Figure 4D* and *E*). Smad1 upregulation and phosphorylation in the aorta of miR-26bKO mice were confirmed and quantified using Western blots (*Figure 4F* and *G*).

Using a luciferase assay for *SMAD1* mRNA 3′UTR, we validated *SMAD1* as a direct target of miR-26b (see Supplementary material online, *Figure S6A*). The regulation of SMAD1 expression by anti-miR-26b and miR-26b mimic was verified in human aortic SMCs (HAoSMCs) (see Supplementary material online, *Figure S6B*). Next, we determined the reciprocal regulation of miR-26b-5p and *SMAD1* during the osteogenic differentiation. For this purpose, HAoSMCs were cultured in high phosphate media (Pi: 2.6 mM) (Hp) or control media for 10 days. miR-26b was downregulated and *SMAD1* upregulated in these conditions (see Supplementary material online, *Figure S6C*), while miR-26b mimics reduced *SMAD1* expression (see Supplementary material online, *Figure S6C*), calcium deposition in HAoSMCs (see Supplementary material online, *Figure S6D*), and ALP activity (see Supplementary material online, *Figure S6E*). Conversely, the knock-down of *SMAD1* (siSMAD1) reduced the expression of *SMAD1* and calcium deposition in HAoSMCs in Hp media (see Supplementary material online, *Figure S6F*–*H*). These results suggest that in miR-26bKO mice, HAoSMCs specifically showed an up-regulation of *SMAD1*, likely leading to the progression of microcalcification.

### 3.5 BMP signalling and aortic cell–cell communication are dysregulated in miR-26bKO mice

**Figure 5:**
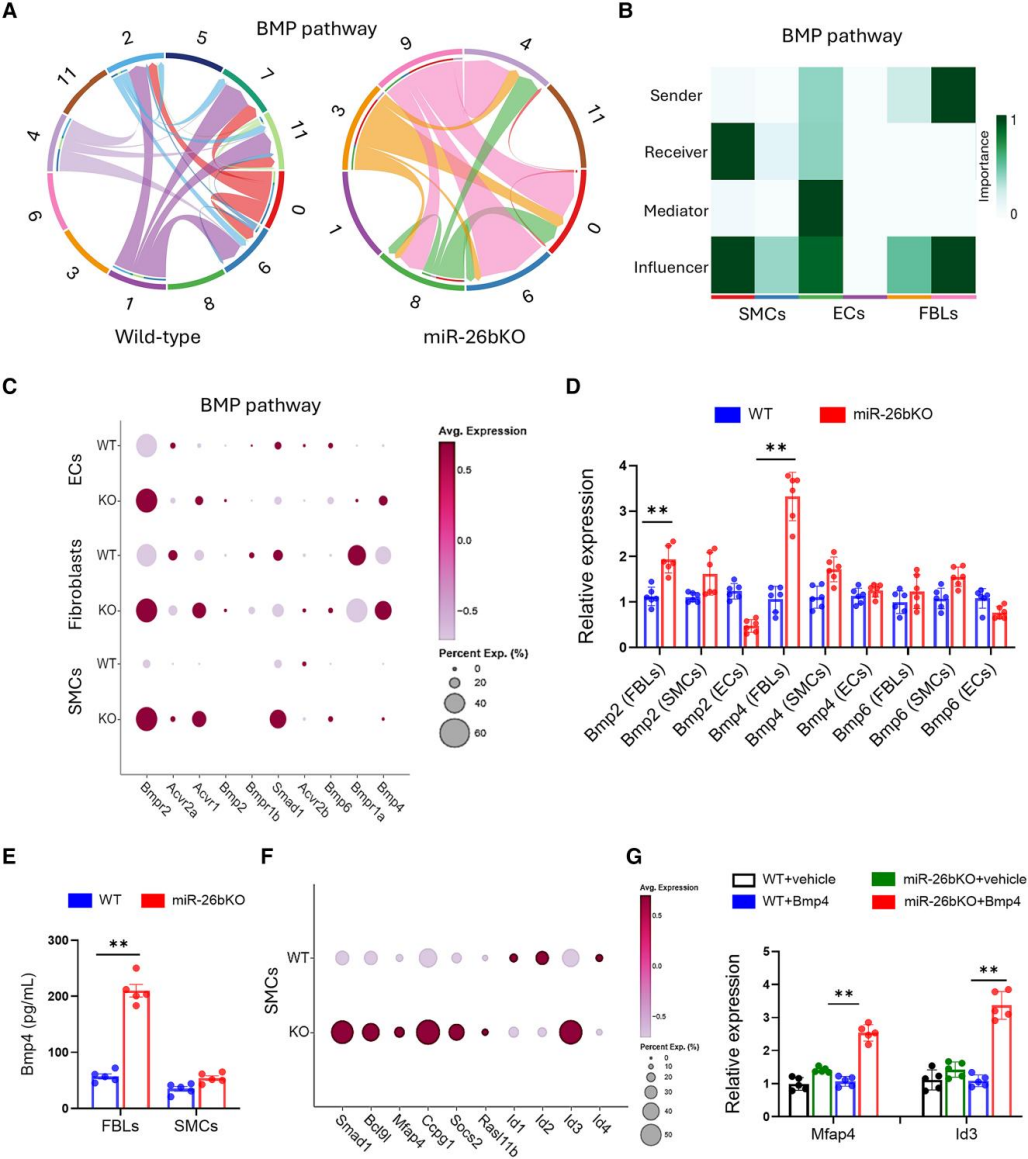
Analysis of cell–cell communication in the aorta of miR-26bKO mice. (*A*) Chord Diagram representing the inferred BMP network: the inner thinner bar colours represent the targets that receive a signal from the outer bar. The inner bar size is proportional to the signal strength received by the targets. Edge colours are consistent with the sources as senders, and edge weights are proportional to the interaction strength. A thicker edge line indicates a stronger signal. (*B*) Signalling roles for BMP pathways in the aorta of miR-26bKO mice in the form of dominant senders, receivers, mediators and influencers for each cell cluster/cell type are inferred from network centrality measures. (*C*) Dot plot presenting average gene expression of BMP pathway target genes in aortic ECs, fibroblasts (FBLs), and smooth muscle cells (SMCs). Average expression is represented by colour, with increased expression represented by a darker colour vs. decreased expression with a lighter colour. Percentage expression (%) is displayed according to the size of the dot, with a larger dot representing a higher percentage of a cell expressing a gene. (*D*) Expression analysis of *Bmp2*, *Bmp4*, and *Bmp6* in isolated aortic SMCs and FBLs from WT and miR-26bKO mice (*n* = 6 per group [3 male/3 female]). (*E*) Bmp4 quantification in WT and miR-26bKO SMC or FBLs media (*n* = 5 per group [3 male/2 female]). (*F*) Dot plot presenting average gene expression of *Smad1* target genes in aortic SMCs from WT and miR-26bKO mice. (*G*) Expression of *Mfap4* and *Id3* in WT and miR-26bKO SMCs treated with Bmp4 for 48 h (*n* = 5 per group [2 male/3 female]). Male and female mice have been used in (*D, E,* and *G*). For (*D* and *E*): **P < 0.01 vs. WT; One-way ANOVA. For (*G*): **P < 0.01 vs. WT + Bmp4; Two-way ANOVA. All data are mean ± SEM.

Upon the binding of BMP ligands to their respective receptors, type II receptors phosphorylate and activate type I receptors, which in turn phosphorylate SMAD1, inducing its nuclear translocation and activating the canonical BMP-SMAD-dependent signalling pathway.^34^ Therefore, we inferred and analysed cell–cell communication pathways from scRNA-seq data^35^ to identify BMP pathway ligand–receptor interactions in the vascular cells that could promote SMAD1 phosphorylation. Differential analysis revealed increased cellular communication among cell clusters in miR-26bKO compared to WT aorta on the BMP pathway (*Figure 5A*). A more detailed analysis demonstrated that FBLs are dominant senders and influencers on the BMP pathways, whereas SMCs are dominant receivers and influencers of the phenotype in miR-26bKO mice (*Figure 5B*). By mapping BMP pathway ligands and receptors to different vascular cells, we demonstrated that miR-26bKO aortic FBLs expressed the BMP ligands, specifically *Bmp4*, which could promote *Smad1* phosphorylation in the SMCs (*Figure 5C*).

To test the validity of the cell–cell communication, we analysed the expression of *Bmp2*, *Bmp4*, and *Bmp6* in isolated aortic SMCs and FBLs from WT and miR-26bKO mice. As predicted, *Bmp4* was the most upregulated BMP ligand in miR-26KO FBLs (*Figure 5D*). Secretion of Bmp4 from miR-26KO FBLs was confirmed by ELISA (*Figure 5E*). To further verify the Smad1-mediated signalling in SMCs from miR-26bKO mice, we retrieved SMAD1 ChIP-seq and RNA-seq data on HAoSMCs stimulated with BMP4.^36^ Then, we mapped the genes whose loci were bounded by SMAD1 and whose expression levels were up-regulated more than two-fold (*Figure 5F*). *Id3* and *Mfap4*, targets of *Smad1* and linked with SMC osteogenic differentiation^37^ and migration,^38^ respectively, were upregulated in the SMC cluster in miR-26bKO. Stimulating miR-26bKO SMC with Bmp4 validated the scRNA-seq data (*Figure 5G*).

### 3.6 Inhibition of ALK receptors blocked cell–cell communication between the aortic cells and reversed microcalcification in the aortas of miR-26bKO mice

**Figure 6:**
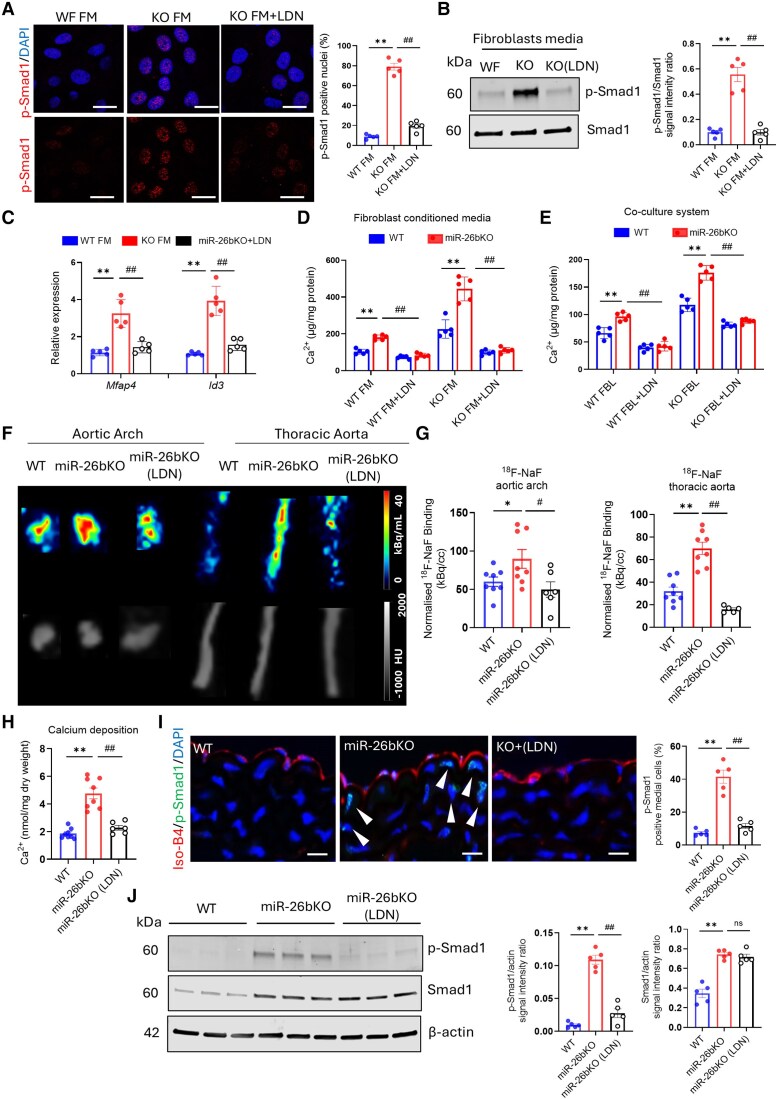
Inhibition of ALK receptors reduces calcium deposition in smooth muscle cells lacking miR-26b and microcalcification in miR-26KO aortas. (*A*) Analysis of p-Smad1 nuclear translocation in miR-26bKO SMCs treated with WT or miR-26bKO FBL-conditioned media in the presence or absence of LDN-193189 (500 nM) for 30 min (*n* = 5 per group [3 male/2 female]). Scale bar: 50 μm. (*B*) Representative Western blots for p-Smad1 (Ser463/465) and total Smad1 in miR-26bKO SMCs treated as above and quantification of signal intensity compared to Smad1 (*n* = 5 per group [3 male/2 female]). (*C*) Expression of *Mfap4* and *Id3* in miR-26bKO SMCs treated with WT or miR-26bKO FBL-conditioned media in the presence or absence of LDN-193189 for 48 h (*n* = 5 per group [3 male/2 female]). (*D*) Analysis of calcium deposition in WT and miR-26bKO SMCs culture in high-phosphate media (2.6 mM). Cells were treated with WT or miR-26bKO FBL-conditioned media in the presence or absence of LDN-193189 for 10 days (*n* = 5 per group [3 male/2 female]). (*E*) Aortic SMCs and FBLs isolated from WT and miR-26bKO mice were co-cultured in high-phosphate media in a Transwell™ with SMCs on the abluminal side of the insert membrane and FBLs on the luminal side. Cells were cultured in the presence or absence of LDN-193189 for 10 days (*n* = 5 per group [3 male/2 female]). (*F*) Representative *ex vivo* micro-PET/CT scans of the heart and descending aorta in wildtype, miR-26bKO mice at 6 months. The colours correspond to the strength of the ^18^F-NaF (hydroxyapatite marker) signal. (*G*) Quantification of microcalcification in the aortic arch and thoracic aorta (*n* = 5 per group). (*H*) Calcium deposition within the mouse aorta (*n* = 6–8 per group). (*I*) Immunostaining of p-Smad1 within the aorta of WT, miR-26bKO and miR-26bKO mice treated with LDN-193189. Scale bar = 100 μm. White arrows: p-Smad1 positive cells and quantification of p-Smad1 positive aortic media cells (*n* = 5 per group). (*J*) Representative Western blots for p-Smad1 (Ser463/465), total Smad1 and β-actin in aortas of WT, miR-26bKO and miR-26bKO mice treated with LDN-193189 and quantification of signal intensity compared to β-actin (loading control) (*n* = 5 per group). For (*A–D*): **P < 0.01 vs. WT FM; ^##^*P* < 0.01 vs. KO FM; For (*E*): **P < 0.01 vs. WT FBLs; ^##^*P* < 0.01 vs. KO FBLs; Two-way ANOVA. Male and female mice have been used in these experiments. For (*G–J*): ns, non-significant; *P < 0.05; **P < 0.01 vs. WT; ^#^*P* < 0.05; ^##^*P* < 0.01 vs. KO; one-way ANOVA. All data are mean ± SEM. Male mice have been used in these experiments.

We then tested whether FBL-conditioned media could activate Smad1 phosphorylation and its nuclear translocation. Mir-26bKO SMCs were stimulated for 30 min with conditioned media from WT or mir-26bKO FBLs (WT FM or KO FM, respectively), and LDN-193189, a potent, selective BMP Type I receptor inhibitor,^39^ was used to block Smad1 phosphorylation and cellular communication. As shown in *Figure 6A*, conditioned media from FBLs isolated from miR-26bKO promoted nuclear translocation of p-Smad1; LDN-193189 inhibited p-Smad1 translocation. Smad-1 phosphorylation and LDN-193189 inhibition in miR-26bKO SMCs stimulated with KO FM was confirmed and quantified by Western blot (*Figure 6B*). Moreover, culturing miR-26bKO SMCs in FBL-conditioned media induced the transcription of *Smad1* target genes (*Id3* and *Mfap4*), which was prevented by LDN-193189 treatment (*Figure 6C*). Previous studies demonstrated that BMPs enhance phosphate-mediated SMC calcification.^40^ To test this in our model, confluent WT and miR-26bKO SMCs were cultured in Hp media in the presence of FBL-conditioned media from WT or miR-26bKO mice for 10 days. LDN-193189 was used to inhibit BMP-dependent calcification in SMCs. As shown in *Figure 6D*, analysis of calcification indicated that (i) miR-26bKO SMCs were more susceptible to calcification than WT SMCs when cultured in high-phosphate media; (ii) miR-26bKO FBL-conditioned media increased matrix mineralization compared to WT FBL-conditioned media. As expected, the treatment with LDN-193189 inhibited calcium accumulation in the SMCs. Furthermore, we exploited a more relevant coculture system in which aortic SMCs and FBLs isolated from WT and miR-26bKO mice were co-cultured in a Transwell™ with SMCs on the abluminal side of the insert membrane and FBLs on the luminal side (see Supplementary material online, *Figure S7A*). The results were consistent with the experiments with FBL-conditioned media, showing that co-culture of miR-26bKO SMCs with miR-26bKO FBLs enhanced calcium accumulation in miR-26bKO SMCs (*Figure 6E*). These results suggest that dysregulated BMP signalling in miR-26bKO cells increases calcium accumulation in SMCs; hence, we investigated the translational potential of SMC-FBL cross-talk inhibition in regulating microcalcification in miR-26bKO mice.

WT and miR-26bKO mice (6-month-old) were randomized to receive an every-other-day injection of LDN-193189 (3 mg/kg; i.p.) or vehicle control for 2 weeks. The treatment of miR-26bKO mice with LDN-193189 resulted in a marked reduction in the level of microcalcification in the aortic arch and thoracic aorta as determined by ^18^F-NaF micro-PET/CT analysis (*Figure 6F* and *G*). We further confirmed the micro-PET/CT data by showing that the increased calcium accumulation in the vessels of miR-26bKO mice was reduced by the LDN-193189 treatment (*Figure 6H*). Histological analysis of the aorta of miR-26bKO mice confirmed that the upregulation of p-Smad1 in the aorta of miR-26bKO mice compared to WT is attenuated by LDN-193189 treatment (*Figure 6I* and Supplementary material online, *Figure S7B*). These results were confirmed and quantified by Western blot (*Figure 6J*).

### 3.7 Inverse correlation of miR-26b and SMAD1 in calcific aortic tissues

**Figure 7:**
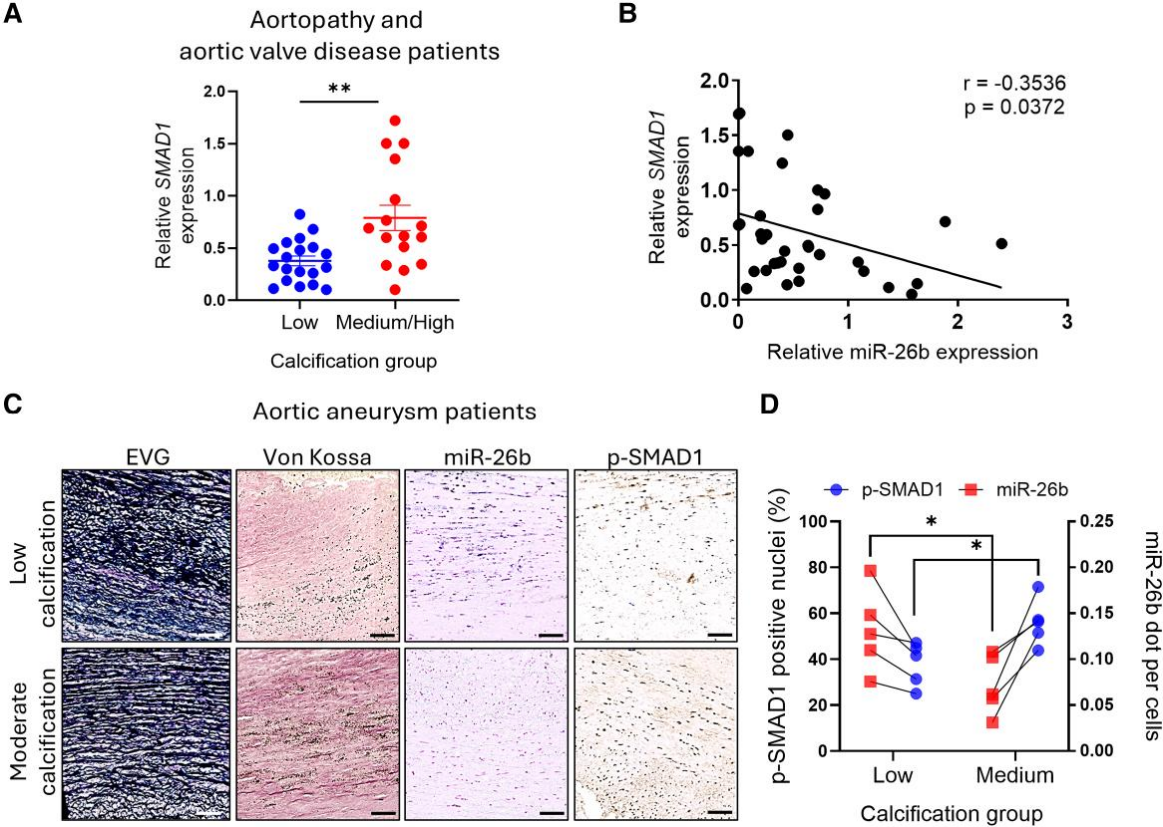
Inverse correlation between miR-26b and SMAD1 in calcific aortic tissue. (*A*) The expression of SMAD1 in low and medium/high calcification samples (*B*) and correlation with the level of miR-26b-5p (*r* = −0.3536, *P* = 0.0372; Spearman correlation). (*C*) *In situ* hybridization for miR-26b-5p, immunostaining of von Kossa and EVG and immunohistochemistry of p-SMAD1 within low and moderately calcified human ascending thoracic aortic aneurysm tissue. Scale bar = 500 μm. (*D*) Quantification of miR-26b-5p positive signals per cell or p-SMAD1 positive signals per nucleus in patient samples with low or moderate microcalcification and association between miR-26b-5p and SMAD1 expression within the same sample. For (*A*) *n* = 19 low (5 female/14 male) and *n* = 16 medium/high (4 female/12 male); *P < 0.05; **P < 0.01 vs. low calcification; Student’s unpaired *t*-test. For (*D*), *P < 0.05 vs. low calcification. Student’s unpaired *t*-test (*n* = 5 per group). All data are mean ± SEM.

*SMAD1* expression was studied in aortic tissues from subgroups of patients with concomitant aortopathy and aortic valve disease (*Figure 1*). *SMAD1* significantly increased across medium/high calcification compared to low calcification samples (*Figure 7A*). In addition, an inverse correlation was found between the expression of miR-26b-5p and *SMAD1* (*Figure 7B*). Further work was undertaken to study miR-26b-5p localization and expression by *in situ* hybridization in another cohort of patients with aortic aneurysms, focusing on low (von Kossa staining 0–0.99%) and medium levels (von Kossa staining < 10%) of calcification with intact elastin.^9^ Analysis of the positive miR-26b-5p probe signal showed that miR-26b-5p localization significantly decreased with increasing levels of mineralization (von Kossa staining) in the aorta, importantly paralleled by a higher expression of p-SMAD1 (*Figure 7C* and Supplementary material online, *Figure S7C* and *D*). Quantitative analyses of histological preparations confirmed the latter observations (*Figure 7D*).

## 4. Discussion

Vascular calcification represents a cardiovascular risk factor for patient morbidity and mortality. It is characterized by a complicated signalling process in which multiple pathways and cell types are involved during various stages of the disease. By integrating single-cell transcriptomics and networking analysis with molecular imaging, we have demonstrated that deletion of miR-26b in mice resulted in spontaneous and age-related aortic microcalcifications by dysregulating cell-specific target genes and cell–cell communication between aortic cells.

miRNAs interact with their target genes’ 3′ untranslated region (3′ UTR) to induce mRNA degradation or translational repression. The interaction is dynamic and dependent on many factors, such as the abundance of miRNAs and target mRNAs, as well as the affinity of miRNA–mRNA interactions. miRNA suppression of mRNA targets is not ubiquitous between cell types. Alternative splicing and alternative polyadenylation affect 3′ UTRs, and cell-type-specific RNA binding proteins may change the miRNA response elements in the target genes, thus making mRNAs sensitive or insensitive to miRNA-mediated gene regulation in a cell-type-specific manner.^41^ Single-cell transcriptomics can facilitate the analysis of cell-specific expression of miRNA target genes and determine miRNA activity.^42,43^ Using this approach, we identified several target genes expressed in aortic cells. We validated our results and found that the miR-26b target gene SMAD1 is specifically expressed in aortic smooth muscle cells (SMCs) in this mouse model.

Differences in targeting approaches in miRNA KO mice, such as lengths of deleted sequences, can also explain the different phenotypes and target GEX. Recent studies using miR-26 family KO mice (CRISPR/Cas9-generated) have shown that only mice lacking all three copies of miR-26 (miR-26a, miR-26a-2, and miR-26b) developed a pathological phenotype, such as an increase in white adipose tissue^44^ or cataracts.^20^ Moreover, the global KO of miR-26b using a floxed conditional mouse model, and therefore using a different deletion approach, revealed dysregulation of genes involved in biological processes related to protein biosynthesis and metabolism in the aorta of KO mice compared to WT.^45^ Hence, our miR-26bKO mouse is a unique model of spontaneous and age-related microcalcification and represents a unique transgenic source for studying the early events in the vascular calcification process, including the different contributions of resident vascular cells to the disease.

Cell cross-talk remains controversial but is a critical aspect in understanding vascular calcification.^46^ Although it was initially believed that the different vessel layers worked independently to maintain homeostasis, recent literature supports a model where communications through extracellular vesicles or soluble factors ensure a healthy vessel microenvironment.^16,47^ The analysis of the BMP pathway in the aortic cells of miR-26bKO mice revealed that FBLs express and release BMP4. Significantly, BMP4 is transcriptionally regulated by GATA4, a key factor in FBLs, during organ development.^48^ BMP4 can promote the phosphorylation of SMAD1 in SMCs. Using FBL-conditioned media and the co-culture model, we dissected the contribution of FBLs to SMC calcium accumulation. Interestingly, miR-26bKO SMCs showed increased calcium accumulation compared to WT when cultured in high-phosphate. Calcium accumulation increased significantly in the presence of FBL media or when SMCs were co-cultured with FBLs, confirming that BMPs could enhance phosphate-mediated SMC calcification.

Microcalcification in miR-26bKO mice was reversible, thus confirming that the early stages of the calcification process represent a potential therapeutic window for pharmacological intervention. With mechanistic insights, therapeutic strategies could be created to target the processes that initiate vascular calcification. In this study, LDN-193189 was used as a lead compound to interfere with the SMC-FBL cross-talk. This compound specifically inhibits BMP receptor-dependent phosphorylation of SMAD1/5/8 while mainly sparing the phosphorylation of SMAD2/3 by the activin and TGF-β receptors.^49^ Drug screening based on LDN-193189 specificity identified Saracatinib, an FDA-approved cancer drug, to target fibrodysplasia ossificans progressiva, a rare disease characterized by ectopic calcification of soft tissues through dysregulation of BMP/SMAD1 pathways, thus revealing a possible therapeutic strategy in patients with mild/moderate aortopathy and microcalcification.^50^

Limitation: It is essential to acknowledge the limitations of our current work. While our study provides valuable insights into the role of miR-26b in vascular calcification, our animal model only represents the early stages of the disease, not the entire spectrum of aortopathy. Additionally, the 6-month follow-up period may not have been sufficient to observe the long-term chronic effect of miR-26b deletion on the vascular calcification process, which is clinically associated with ageing and other cardiovascular risk factors. Furthermore, this study focuses on the cross-talk signalling between FBLs and SMCs, although other potential signalling pathways, such as those involving ECs and immune cells, could be involved. Nevertheless, our mouse model offers a platform for developing additional studies by including relevant cardiovascular risk factors.

In conclusion, this investigation has demonstrated the contribution of miR-26b in initiating vascular calcification. Integrating transcriptomics, molecular imaging, and network analysis, we created a complete gene-to-pathway understanding of the FBL-SMC cross-talk leading to SMC calcification. Using samples from patients with vascular disease, we demonstrated that similar processes mediated by miR-26b downregulation potentially operate in conditions where vascular calcification is a common pathological feature associated with poor prognosis and severity. Therefore, these findings reveal critical mechanisms in regulating calcification initiation *in vivo* and suggest new therapeutic targets for aortic disease.

## Supplementary Material

cvaf117_Supplementary_Data

## Data Availability

The RNA-sequencing datasets generated during the study are available in the Gene Expression Omnibus repository (http://www.ncbi.nlm.nih.gov/geo/) with the following accession numbers: GSE281028 (scRNA-seq) and GSE281279 (bulk RNA-seq).

## References

[1] Demer LL, Tintut Y. Vascular calcification: pathobiology of a multifaceted disease. Circulation 2008;117:2938–2948. 18519861 10.1161/CIRCULATIONAHA.107.743161 PMC4431628

[2] Strauss HW, Nakahara T, Narula N, Narula J. Vascular calcification: the evolving relationship of vascular calcification to major acute coronary events. J Nucl Med 2019;60:1207–1212. 31350320 10.2967/jnumed.119.230276

[3] McClelland RL, Chung H, Detrano R, Post W, Kronmal RA. Distribution of coronary artery calcium by race, gender, and age: results from the Multi-Ethnic Study of Atherosclerosis (MESA). Circulation 2006;113:30–37. 16365194 10.1161/CIRCULATIONAHA.105.580696

[4] Rennenberg RJ, Kessels AG, Schurgers LJ, van Engelshoven JM, de Leeuw PW, Kroon AA. Vascular calcifications as a marker of increased cardiovascular risk: a meta-analysis. Vasc Health Risk Manag 2009;5:185–197. 19436645 10.2147/vhrm.s4822 PMC2672434

[5] Pawade TA, Newby DE, Dweck MR. Calcification in aortic stenosis: the skeleton key. J Am Coll Cardiol 2015;66:561–577. 26227196 10.1016/j.jacc.2015.05.066

[6] Joshi NV, Vesey AT, Williams MC, Shah AS, Calvert PA, Craighead FH, Yeoh SE, Wallace W, Salter D, Fletcher AM, van Beek EJ, Flapan AD, Uren NG, Behan MW, Cruden NL, Mills NL, Fox KA, Rudd JH, Dweck MR, Newby DE. 18F-fluoride positron emission tomography for identification of ruptured and high-risk coronary atherosclerotic plaques: a prospective clinical trial. Lancet 2014;383:705–713. 24224999 10.1016/S0140-6736(13)61754-7

[7] Irkle A, Vesey AT, Lewis DY, Skepper JN, Bird JL, Dweck MR, Joshi FR, Gallagher FA, Warburton EA, Bennett MR, Brindle KM, Newby DE, Rudd JH, Davenport AP. Identifying active vascular microcalcification by (18)F-sodium fluoride positron emission tomography. Nat Commun 2015;6:7495. 26151378 10.1038/ncomms8495 PMC4506997

[8] Forsythe RO, Dweck MR, McBride OMB, Vesey AT, Semple SI, Shah ASV, Adamson PD, Wallace WA, Kaczynski J, Ho W, van Beek EJR, Gray CD, Fletcher A, Lucatelli C, Marin A, Burns P, Tambyraja A, Chalmers RTA, Weir G, Mitchard N, Tavares A, Robson JMJ, Newby DE. (18)F-sodium fluoride uptake in abdominal aortic aneurysms: the SoFIA(3) study. J Am Coll Cardiol 2018;71:513–523. 29406857 10.1016/j.jacc.2017.11.053 PMC5800891

[9] Fletcher AJ, Nash J, Syed MBJ, Macaskill MG, Tavares AAS, Walker N, Salcudean H, Leipsic JA, Lim KHH, Madine J, Wallace W, Field M, Newby DE, Bouchareb R, Seidman MA, Akhtar R, Sellers SL. Microcalcification and thoracic aortopathy: a window into disease severity. Arterioscler Thromb Vasc Biol 2022;42:1048–1059. 35770666 10.1161/ATVBAHA.122.317111 PMC9311465

[10] Hortells L, Sur S, St Hilaire C. Cell phenotype transitions in cardiovascular calcification. Front Cardiovasc Med 2018;5:27. 29632866 10.3389/fcvm.2018.00027 PMC5879740

[11] Durham AL, Speer MY, Scatena M, Giachelli CM, Shanahan CM. Role of smooth muscle cells in vascular calcification: implications in atherosclerosis and arterial stiffness. Cardiovasc Res 2018;114:590–600. 29514202 10.1093/cvr/cvy010 PMC5852633

[12] Li W, Su SA, Chen J, Ma H, Xiang M. Emerging roles of fibroblasts in cardiovascular calcification. J Cell Mol Med 2021;25:1808–1816. 33369201 10.1111/jcmm.16150 PMC7882970

[13] Blaser MC, Kraler S, Luscher TF, Aikawa E. Multi-omics approaches to define calcific aortic valve disease pathogenesis. Circ Res 2021;128:1371–1397. 33914608 10.1161/CIRCRESAHA.120.317979 PMC8095729

[14] O'Brien J, Hayder H, Zayed Y, Peng C. Overview of MicroRNA biogenesis, mechanisms of actions, and circulation. Front Endocrinol (Lausanne) 2018;9:402. 30123182 10.3389/fendo.2018.00402 PMC6085463

[15] Inui M, Martello G, Piccolo S. MicroRNA control of signal transduction. Nat Rev Mol Cell Biol 2010;11:252–263. 20216554 10.1038/nrm2868

[16] Goettsch C, Hutcheson JD, Aikawa E. MicroRNA in cardiovascular calcification: focus on targets and extracellular vesicle delivery mechanisms. Circ Res 2013;112:1073–1084. 23538277 10.1161/CIRCRESAHA.113.300937 PMC3668680

[17] Zhang Y, Xie RL, Croce CM, Stein JL, Lian JB, van Wijnen AJ, Stein GS. A program of microRNAs controls osteogenic lineage progression by targeting transcription factor Runx2. Proc Natl Acad Sci U S A 2011;108:9863–9868. 21628588 10.1073/pnas.1018493108 PMC3116419

[18] Icli B, Dorbala P, Feinberg MW. An emerging role for the miR-26 family in cardiovascular disease. Trends Cardiovasc Med 2014;24:241–248. 25066487 10.1016/j.tcm.2014.06.003 PMC4150842

[19] Martello A, Mellis D, Meloni M, Howarth A, Ebner D, Caporali A, Al Haj Zen A. Phenotypic miRNA screen identifies miR-26b to promote the growth and survival of endothelial cells. Mol Ther Nucleic Acids 2018;13:29–43. 30227275 10.1016/j.omtn.2018.08.006 PMC6141730

[20] Upreti A, Hoang TV, Li M, Tangeman JA, Dierker DS, Wagner BD, Tsonis PA, Liang C, Lachke SA, Robinson ML. miR-26 deficiency causes alterations in lens transcriptome and results in adult-onset cataract. Invest Ophthalmol Vis Sci 2024;65:42. 10.1167/iovs.65.4.42 PMC11059818 38683565

[21] Schmittgen TD, Livak KJ. Analyzing real-time PCR data by the comparative C(T) method. Nat Protoc 2008;3:1101–1108. 18546601 10.1038/nprot.2008.73

[22] Hu D, Yin C, Mohanta SK, Weber C, Habenicht AJ. Preparation of single cell suspensions from mouse aorta. Bio Protoc 2016;6:e1832. 10.21769/bioprotoc.1832 PMC4913879 27335895

[23] He D, Patro R. Simpleaf: a simple, flexible, and scalable framework for single-cell data processing using alevin-fry. Bioinformatics 2023;39:btad614. 37802884 10.1093/bioinformatics/btad614 PMC10580267

[24] Jiang A, Lehnert K, You L, Snell RG. ICARUS, an interactive web server for single cell RNA-Seq analysis. Nucleic Acids Res 2022;50:W427–W433. 35536286 10.1093/nar/gkac322 PMC9252722

[25] Jin S, Plikus MV, Nie Q. CellChat for systematic analysis of cell-cell communication from single-cell transcriptomics. Nat Protoc 2024;50:W427–W433. 10.1038/s41596-024-01045-4 39289562

[26] Shannon P, Markiel A, Ozier O, Baliga NS, Wang JT, Ramage D, Amin N, Schwikowski B, Ideker T. Cytoscape: a software environment for integrated models of biomolecular interaction networks. Genome Res 2003;13:2498–2504. 14597658 10.1101/gr.1239303 PMC403769

[27] Tastsoglou S, Skoufos G, Miliotis M, Karagkouni D, Koutsoukos I, Karavangeli A, Kardaras FS, Hatzigeorgiou AG. DIANA-miRPath v4.0: expanding target-based miRNA functional analysis in cell-type and tissue contexts. Nucleic Acids Res 2023;51:W154–W159. 37260078 10.1093/nar/gkad431 PMC10320185

[28] McGeary SE, Lin KS, Shi CY, Pham TM, Bisaria N, Kelley GM, Bartel DP. The biochemical basis of microRNA targeting efficacy. Science 2019;366:eaav1741. 31806698 10.1126/science.aav1741 PMC7051167

[29] Garaikoetxea M, Martin-Nunez E, Navarro A, Matilla L, Fernandez-Celis A, Arrieta V, Garcia-Pena A, Gainza A, Alvarez V, Sadaba R, Jover E, Lopez-Andres N. Targeting fatty acid-binding protein 4 improves pathologic features of aortic stenosis. Int J Mol Sci 2022;23:8439. 35955575 10.3390/ijms23158439 PMC9369247

[30] Kalluri AS, Vellarikkal SK, Edelman ER, Nguyen L, Subramanian A, Ellinor PT, Regev A, Kathiresan S, Gupta RM. Single-cell analysis of the normal mouse aorta reveals functionally distinct endothelial cell populations. Circulation 2019;140:147–163. 31146585 10.1161/CIRCULATIONAHA.118.038362 PMC6693656

[31] Ord T, Lonnberg T, Nurminen V, Ravindran A, Niskanen H, Kiema M, Ounap K, Maria M, Moreau PR, Mishra PP, Palani S, Virta J, Liljenback H, Aavik E, Roivainen A, Yla-Herttuala S, Laakkonen JP, Lehtimaki T, Kaikkonen MU. Dissecting the polygenic basis of atherosclerosis via disease-associated cell state signatures. Am J Hum Genet 2023;110:722–740. 37060905 10.1016/j.ajhg.2023.03.013 PMC10183377

[32] Chakraborty A, Li Y, Zhang C, Li Y, Rebello KR, Li S, Xu S, Vasquez HG, Zhang L, Luo W, Wang G, Chen K, Coselli JS, LeMaire SA, Shen YH. Epigenetic induction of smooth muscle cell phenotypic alterations in aortic aneurysms and dissections. Circulation 2023;148:959–977. 37555319 10.1161/CIRCULATIONAHA.123.063332 PMC10529114

[33] Blaser MC, Kraler S, Luscher TF, Aikawa E. Network-guided multiomic mapping of aortic valve calcification. Arterioscler Thromb Vasc Biol 2023;43:417–426. 36727519 10.1161/ATVBAHA.122.318334 PMC9975082

[34] Yang P, Troncone L, Augur ZM, Kim SSJ, McNeil ME, Yu PB. The role of bone morphogenetic protein signaling in vascular calcification. Bone 2020;141:115542. 32736145 10.1016/j.bone.2020.115542 PMC8185454

[35] Jin S, Guerrero-Juarez CF, Zhang L, Chang I, Ramos R, Kuan CH, Myung P, Plikus MV, Nie Q. Inference and analysis of cell-cell communication using CellChat. Nat Commun 2021;12:1088. 33597522 10.1038/s41467-021-21246-9 PMC7889871

[36] Morikawa M, Koinuma D, Tsutsumi S, Vasilaki E, Kanki Y, Heldin CH, Aburatani H, Miyazono K. ChIP-seq reveals cell type-specific binding patterns of BMP-specific Smads and a novel binding motif. Nucleic Acids Res 2011;39:8712–8727. 21764776 10.1093/nar/gkr572 PMC3203580

[37] Huang A, Rao J, Feng X, Li X, Xu T, Yao L. Breaking new ground: unraveling the USP1/ID3/E12/P21 axis in vascular calcification. Transl Res 2024;276:1–20. 39326697 10.1016/j.trsl.2024.09.002

[38] Schlosser A, Pilecki B, Hemstra LE, Kejling K, Kristmannsdottir GB, Wulf-Johansson H, Moeller JB, Fuchtbauer EM, Nielsen O, Kirketerp-Moller K, Dubey LK, Hansen PB, Stubbe J, Wrede C, Hegermann J, Ochs M, Rathkolb B, Schrewe A, Bekeredjian R, Wolf E, Gailus-Durner V, Fuchs H, Hrabe de Angelis M, Lindholt JS, Holmskov U, Sorensen GL. MFAP4 promotes vascular smooth muscle migration, proliferation and accelerates neointima formation. Arterioscler Thromb Vasc Biol 2016;36:122–133. 26564819 10.1161/ATVBAHA.115.306672

[39] Cuny GD, Yu PB, Laha JK, Xing X, Liu JF, Lai CS, Deng DY, Sachidanandan C, Bloch KD, Peterson RT. Structure-activity relationship study of bone morphogenetic protein (BMP) signaling inhibitors. Bioorg Med Chem Lett 2008;18:4388–4392. 18621530 10.1016/j.bmcl.2008.06.052 PMC2570262

[40] Li X, Yang HY, Giachelli CM. BMP-2 promotes phosphate uptake, phenotypic modulation, and calcification of human vascular smooth muscle cells. Atherosclerosis 2008;199:271–277. 18179800 10.1016/j.atherosclerosis.2007.11.031 PMC3249145

[41] Nam JW, Rissland OS, Koppstein D, Abreu-Goodger C, Jan CH, Agarwal V, Yildirim MA, Rodriguez A, Bartel DP. Global analyses of the effect of different cellular contexts on microRNA targeting. Mol Cell 2014;53:1031–1043. 24631284 10.1016/j.molcel.2014.02.013 PMC4062300

[42] Herbst E, Mandel-Gutfreund Y, Yakhini Z, Biran H. Inferring single-cell and spatial microRNA activity from transcriptomics data. Commun Biol 2025;8:87. 39827321 10.1038/s42003-025-07454-9 PMC11743151

[43] Maji RK, Leisegang MS, Boon RA, Schulz MH. Revealing microRNA regulation in single cells. Trends Genet 2025;41:522–536. 39863489 10.1016/j.tig.2024.12.009

[44] Acharya A, Berry DC, Zhang H, Jiang Y, Jones BT, Hammer RE, Graff JM, Mendell JT. miR-26 suppresses adipocyte progenitor differentiation and fat production by targeting Fbxl19. Genes Dev 2019;33:1367–1380. 31488578 10.1101/gad.328955.119 PMC6771383

[45] van der Vorst EPC, Pepe MAA, Peters LJF, Haberbosch M, Jansen Y, Naumann R, Stathopoulos GT, Weber C, Bidzhekov K. Transcriptome signature of miRNA-26b KO mouse model suggests novel targets. BMC Genom Data 2021;22:23. 34193044 10.1186/s12863-021-00976-1 PMC8243710

[46] Yang S, Zeng Z, Yuan Q, Chen Q, Wang Z, Xie H, Liu J. Vascular calcification: from the perspective of crosstalk. Mol Biomed 2023;4:35. 37851172 10.1186/s43556-023-00146-y PMC10584806

[47] Bardeesi ASA, Gao J, Zhang K, Yu S, Wei M, Liu P, Huang H. A novel role of cellular interactions in vascular calcification. J Transl Med 2017;15:95. 28464904 10.1186/s12967-017-1190-z PMC5414234

[48] Nemer G, Nemer M. Transcriptional activation of BMP-4 and regulation of mammalian organogenesis by GATA-4 and -6. Dev Biol 2003;254:131–148. 12606287 10.1016/s0012-1606(02)00026-x

[49] Yu PB, Hong CC, Sachidanandan C, Babitt JL, Deng DY, Hoyng SA, Lin HY, Bloch KD, Peterson RT. Dorsomorphin inhibits BMP signals required for embryogenesis and iron metabolism. Nat Chem Biol 2008;4:33–41. 18026094 10.1038/nchembio.2007.54 PMC2727650

[50] Williams E, Bagarova J, Kerr G, Xia DD, Place ES, Dey D, Shen Y, Bocobo GA, Mohedas AH, Huang X, Sanderson PE, Lee A, Zheng W, Economides AN, Smith JC, Yu PB, Bullock AN. Saracatinib is an efficacious clinical candidate for fibrodysplasia ossificans progressiva. JCI Insight 2021;6:e95042. 33705358 10.1172/jci.insight.95042 PMC8119212

