# Supplementary material for: The loss of microRNA-26b promotes aortic calcification through the regulation of cell-specific target genes
Source: Cardiovasc Res. 2025 Jul 30;121(11):1778–92. doi: 10.1093/cvr/cvaf117 (PMC12477678; doi:10.1093/cvr/cvaf117)
Supplement: cvaf117_Supplementary_Data [file cvaf117_supplementary_data.pdf]

SUPPLEMENTARY MATERIALS

Extended Materials and Methods

Figures S1 to S7

Tables S1 to S4

References

EXTENDED MATERIALS AND METHODS

Human ascending aortic tissue from patients with aneurysm

Specimens of aneurysmal thoracic aortic tissue were obtained from the Royal Infirmary of Edinburgh, United Kingdom. Samples were collected from consenting patients or relatives under ethical approval (15/ES/0094). Aortic aneurysm specimen preparation and histological characterisation were previously reported in[1]. Briefly, specimens were obtained at the time of surgical repair. Aortic tissue from patients with a dissection was taken from the entry tear or the nearby true lumen. Samples were immediately fixed in 4% paraformaldehyde for at least 24 hours before a five-µm sample of aneurysmal aortic tissue was cut and paraffin-embedded in the axial plane. Sections were embedded in the axial plane to generate an aortic wall cross-section. The clinical and demographic characteristics of the enrolled patients are described in[1].

Human ascending aortic tissue from patients with concomitant aortopathy and aortic valve disease

Specimens of ascending aortic tissues were obtained from patients diagnosed with severe aortic valve disease (e.g., aortic regurgitation or aortic stenosis) and aortopathy elective of concomitant surgical aortic valve and aorta replacement (e.g., Bentall procedure), as well as patients with lone aortopathy elective for valve-sparing aortic root replacement (e.g., David procedure) at Hospital Universitario de Navarra, Spain. The study was covered by the Research Ethics Committee approval (Pyto. 2013/26, num 137) in agreement with the Spanish law (BPCCPMP/ICH/135/95) and according to the ethical principles recorded in the 1975 Declaration of Helsinki and later amendments. All recruited patients provided informed written consent. All patients underwent preoperative transthoracic or transoesophageal echocardiography, according to the clinician's criterion. Peripheral blood samples were collected 24 hours before the surgery for routine biochemical analyses. Exclusion criteria were concomitant mitral valve disease, diabetes, endocarditis, chronic kidney disease, malignant tumour, or other chronic inflammatory disease. Harvested aortas were in 10% neutral buffered formalin for ≥ 24h and paraffin-embedded in the axial plane. The clinical and demographic characteristics of the enrolled patients are described in <u>Table S1</u>.

miR-26bKO generation

The global miR-26b knock-out (KO) mouse was generated using CRISPR/Cas9 via FVB/N zygote microinjection[2]. Two specific guide RNAs (gRNAs) flanking each miRNA genomic sequence were designed using the CRISPR design tool (http://crispr.mit.edu/) and synthesised via gBlocks Gene Fragment (IDT Integrated DNA Technologies, Coralville, IA, USA). gRNAs were *in vitro* transcribed using the in vitro MEGAscript T7 transcription kit (ThermoFisher Scientific) and purified using the MEGAclear Transcription Clean-up kit ThermoFisher Scientific). A mixture of Cas9 mRNA (50 ng/μL; TriLink Biotechnologies) and two specific gRNAs (25 ng/μL each) for each miRNA target was injected into single-cell zygotes. Desired miR-26bKO mice were screened by PCR and confirmed by DNA sequencing and RT-qPCR (primers and gRNA sequences listed in Supplementary Table S2). To test the off-target effects of the gRNAs, for each gRNA, the top four genes with the highest risk of being targeted in the exon regions in founder mice were analysed and confirmed by PCR and DNA sequencing. miR-26bKO mice were backcrossed in C57BL/6J background and were bred and maintained at the University of Edinburgh.

***In vivo* Study**

All experiments involving mice were performed following the guidance and operation of the Animals (Scientific Procedures) Act 1986 and the prior approval of the UK Home Office and the University of Edinburgh Animal Welfare and Ethical Review Board. All animals were housed at the Little France Facility in a dedicated pathogen-free animal facility with 12-hour light/12-hour dark cycles and ad libitum access to food and water. WT and miR-26bKO mice received either LDN-193189 (i.p. 3 mg/kg/d) or vehicle (DMSO) every second day for 14 days (n = 8/group). The endpoints were the levels of calcification and histological analysis. For in vivo studies, the sample size and the sex for each experiment are indicated in the figure legend.

Cell Culture and reagents.

Human Aortic Smooth Muscle Cells (HAoSMCs) were purchased from PromoCell (C-12533) and grown in Smooth Muscle Cell Growth Medium 2 (C-22211, Promocell) supplemented with a Growth Medium 2 SupplementPack (C-22062, Promocell). The medium was changed every two days. According to the manufacturer's instructions, HASMCs were transfected with lipofectamine RNAiMAX (ThermoFisher Scientific), miR-26b-5p mimics, anti-miR-26b-5p and control oligonucleotides (final concentration of 50 nM). LDN-193189 dihydrochloride (500 mM) was purchased from Tocris (Cat. No. 6053). ELISA for mouse Bmp4 was purchased from Novus Biologicals (NBP2-82123).

Aortic cells isolation

Primary fibroblasts and smooth muscle cells were obtained from adult C57BL/6 animals. Aortas were cleaned from periaortic fat, adventitia was separated mechanically from media, and the obtained layers were placed separately into enzyme solution (Collagenase II, Elastase, Soybean Trypsin Inhibitor in HBSS with calcium and magnesium) for 1 hour at 37°C in 5% CO2 in the incubator. Cells were cultured in DMEM (Gibco) supplemented with 10%FBS until 90% confluent. Primary mouse fibroblast cells were obtained from 8-10 weeks-old C57BL/6 animals. Aortas were cleaned from periaortic fat, and adventitia was mechanically separated from the media and placed into separate enzyme solutions (Collagenase II 1mg/mL, Elastase 0.744 unit/mL, Soybean Trypsin Inhibitor 1mg/mL in HBSS with calcium and magnesium) for

1 hour at 37°C in incubator. Cells were cultured in DMEM/F12 (Gibco) supplemented with 20%FBS until 90% confluent. Endothelial cell suspensions were stained for CD31 and CD45 antibodies, washed and re-suspended in PBS at 4 °C and analysed using a BD 5 L LSR Fortessa and BD FACSDiva software (BD Biosciences). To exclude dead cells, DAPI was added before flow cytometry analysis. Cells were sorted using a FACS Aria II instrument and BD FACSDiva software (BD Biosciences).

High-phosphate protocol and Calcium and Alkaline Phosphatase assays

For the high-phosphate protocol (Hp), HAoSMCs or mouse-isolated SMCs were cultured in M199 (cat 12340030, Gibco) supplemented with 20% FBS. At 90% of confluence, cells were starved for 16-24h in M199 supplemented with 0.4% FBS. Then, cells were cultured in Hp calcific (M199 supplemented with 4% FBS, 2.6mM phosphate buffer [stock at 0.2M $Na_2HPO_4{\cdot}2H_2O$ + $NaH_2PO_4{\cdot}H_2O$) or control media for 10 days. Media was replaced every 3 days. Samples were washed with PBS, and calcium was extracted with 0.6N HCl at 4° for 16 hours. Calcium hydroacidic extracts were collected, centrifuged at 10,000 g and transferred to a new tube for calcium analysis by the *o*-cresolphthalein method following the Calcium Colorimetric Assay Kit (MAK022, Sigma-Aldrich) instructions. Cells were lysed with 0.1% SDS/0.1M NaOH, and BCA protein content was performed to normalise calcium amounts on protein content. For the ALP activity assay, cells were washed in cold PBS, resuspended in an Assay Buffer and homogenised using a Dounce homogeniser on ice. Samples were centrifuged at 4°C at top speed for 15 minutes in a cold microcentrifuge to remove insoluble material. The supernatant was collected and transferred to a new tube. ALP activity was analysed following Alkaline Phosphatase Assay kit instructions (MAK447, Sigma-Aldrich).

Co-culture system

Aortic SMCs and FBLs isolated from WT and miR-26bKO male and female mice were cocultured in a Transwell (0.4 μm) with SMCs on the abluminal side of the insert membrane and FBLs on the luminal side. The cells were cultured in Hp media for 10 days in the presence or absence of LDN-193189 (500 mM)[3]. Then, the FBL layer was removed by scraping the cells, and calcium deposition was analysed in the SMC layer using the Calcium Colorimetric Assay Kit (MAK022) as described above.

Histology

Five μm sections were deparaffinised and subsequently hydrated to distilled water. Sample calcification was assessed by staining with 2% (w/v) Alizarin Red aqueous solution for 3 min. Alizarin Red solution pH was adjusted to pH 4.1–4.3 with 10% NH_4OH (A5533; Merck/Sigma-Aldrich, UK). Samples were then dehydrated in acetone, acetone:xylene (1:1) and xylene. Elastin synthesis was proven using EVG staining. Fresh Verhoeff's working solution was prepared with 2.5:1:1 of 5% (w/v) alcoholic hematoxylin, 10% (w/v) aqueous ferric chloride and Weigert's iodine solution. Samples were incubated for up to 1h, rinsed twice in tap water and differentiated in 2% (v/v) aqueous ferric chloride for 2 min. Samples were then incubated with 5% (w/v) sodium thiosulfate for 5 min and counterstained in van Giesson's solution (1% (v/v) aqueous acid fuchsin in saturated picric acid. Sirius Red staining was performed to analyse the whole collagen content. Sirius Red/Direct Red 80 solution was prepared at 0.1% in picric acid and incubated for 30 min. All reagents were provided by

Merck/Sigma-Aldrich (UK). Bright-field or polarised lenses were used to image the stained AVs.

Images analysis: Digital image analyses were additionally performed for histopathological characterisation. In brief, arbitrary fields per section were imaged at 50 or 400X magnification, as appropriate. The content of molecular and histological targets (*e.g.*, thin and thick collagen fibers, calcium deposits or mature bone, fibrosis, and elastic fibers content) were quantified using Image J software. Images were preprocessed to remove background noise or artefacts on the surrounding areas of the aortic rings. Positive ROI were detected by RGB filtering. A subsequent binary conversion of the images on the appropriate channel was performed to quantify the positive % of the area occupied by the targets mentioned above, as previously published[3]. For example, green channel images were used to analyse bright field positive Sirius Red areas, Alizarin Red or thick fibers in polarised light Sirus Red; blue channel for thin fibers in polarised light Sirus Red images; or red channel for elastic fibers (EVG). Manual corrections were applied only when necessary (*e.g*., incomplete or incorrect automatic detection). All quantifications were normalised to the area of the tissue analysed. A blinded approach was implemented for histological analysis.

RNA isolation, RT and qPCR

Total RNA was isolated according to a standardised phenol-chloroform protocol, using Qiazol reagent and miRNeasy miniKit (217004, QIAGEN, Germany), and reverse-transcribed into single-stranded cDNA, using an iScript Advanced cDNA Synthesis Kit (Bio-Rad) for mRNA analyses. Downstream qPCR amplification was performed using iQ SYBR Green Supermix (Bio-Rad) in a CFX Connect Real-Time PCR System (Bio-Rad) using primers at 300mM final concentration. The validated primers are commercially available from Sigma (KICqStartTM Primers). *GAPDH, ACTA2, 18S*, and *HRPT* were used as housekeeping genes.

To study the expression of miR-26b-5p, specific Taqman microRNA assay primers and probes were used (TaqMan™ microRNA Control Assay U6 snRNA, Cat #4427975, assay ID: 001973 and Taqman™ probe miR-26b-5p, Cat #4427975, assay ID:000407, respectively) Reverse transcription was performed with the TaqMan™ MicroRNA Reverse Transcription Kit (Applied Biosystems) using 10ng total RNA. Downstream qPCR amplifications of first-strand cDNA were performed using TaqMan® Universal PCR Master Mix, no AmpErase® UNG(4324018). U6 snRNA was used as a housekeeping gene for miRNA determinations. The relative expression of each selected gene product was calculated using the $2^{-\Delta\Delta Ct}$ method [4]. All reactions were performed in technical triplicates.

Ex-vivo ^{18}F-NaF micro-PET/CT

The whole mouse aorta was harvested from 3 and 6-month-old wild-type and miR-26bKO male and female mice. The aorta was dissected from surrounding tissue, and the perivascular fat was removed. Aortas were incubated with 103–127 kBq/mL 18F-NaF in PBS for 30 min and washed twice in PBS. The tissue was then placed in the scanner, and a 30-minute PET scan was performed using a 3-dimensional 1:5 mode. A CT scan (semi-circular full trajectory, maximum field of view, 720 projections, 50 kVp, 300 ms and 1:4 binning) was acquired for attenuation correction and quantification of microcalcification. Scans were reconstructed using Mediso's iterative Tera-Tomo 3D reconstruction algorithm. PET data was analysed using PMOD version 4.2 (PMOD

Technologies, Switzerland). Briefly, volumes of interest were drawn around the aortic arch and thoracic aorta. The mean signal across the 10 highest-intensity voxels within that volume of interest was then calculated using the hot average function and normalised to the target concentration of 100 kBq/ml. PET images were created by scaling the images using the radiotracer incubation concentration relative to the target concentration and applying a 0.5 x 0.5 x 0.5 Gaussian filter.

Echocardiography
Echocardiography was performed using a Vevo 3100 Imaging System with a 40-MHz linear probe (VisualSonics, Amsterdam). Before scanning, mice were anaesthetised via inhalation of 4% isoflurane at induction (Merial Animal Health Limited) in medical oxygen (BOC Medical, Manchester, UK). Core body temperature was maintained at 37°C. The body temperature of mice was continuously monitored using a rectal probe. Anaesthesia was monitored and maintained by 1.5% isoflurane through inhalation. Aqueous ultrasound solution (Aquasonic 100, Parker Laboratories INC) was applied to the abdomen of mice to facilitate taking readings. A standard 2D echocardiographic study was performed in the parasternal long-axis and short-axis views to assess LV dimensions and systolic function. After scanning, mice were transferred to recovery cages on a heated plate until consciousness was regained. Analysis of ultrasound data was completed using VisualSonics VevoLab 3100 Version 5.7.1 software (FUJIFILM, VisualSonics, Amsterdam).

MicroRNA-26b *in situ* hybridisation
Tissues were embedded in paraffin cut into 6µm thick sections and mounted on Superfrost glass slides. Briefly, slides were baked at 55°C overnight, dewaxed, rehydrated and air dried. Slides were then fixed with 4% PFA at room temperature (RT) for 20 minutes before being treated with proteinase K (1mg/ml) (3115879001, Roche) at 37°C for 12 minutes. Following treatment, slides were then fixed again in 4% PFA before being rinsed in DEPC-water to allow treatment of pre-hybridisation solution (K2191050, Amsbio, UK). Slides were then hybridised overnight using a hybridisation solution (K2191050, Amsbio, UK) at 45°C using 80nM detection digoxigenin probes (miRCURY LNA miRNA Detection probe, Qiagen, Germany). Post hybridisation, slides were washed (10 minutes in 2xSSC, 10 minutes in 1.5xSSC at 45°C and twice for 0.2xSSC at 37°C for 20 minutes each) before placing in blocking solution (K2191050, Amsbio, UK) at RT for 1 hour. After blocking, slides were incubated in alkaline phosphatase-conjugated anti-digoxigenin antibody (1:100 dilution) (K2191050, Amsbio, UK) for 4 hours at 4°C. After 3 washes in PBS for 10 minutes each, alkaline phosphatase buffer was placed for 5 minutes at RT. NBT/BCIP solution (11681451001, Roche, Germany) was added to the slides overnight. Slides were washed in distilled water before counterstaining with nuclear fast red solution (N3020, Sigma, USA). Slides were dehydrated and cleared in xylene, allowing for mounting using the Eukitt mounting medium. Tissues were then imaged using Axio slide scan Z.1 (Zeiss, Oberkochen, Germany)—negative control images are displayed in Supplementary Figure 7.

Immunohistochemistry
DAB staining for SMAD1 (pSMAD1/5, Invitrogen #700047) was performed using the Leica Bond III autostainer (Leica Biosystems, United States) and Bond Polymer Refine Detection kit (DS9800, Lecia Biosystems, United States). Briefly, slides were deparaffinised in xylene and rehydrated through serial dilutions of alcohol to washes in distilled water. Tissues were then imaged using Axio Scan Z.1 (Zeiss, Oberkochen, Germany). Representative negative control images consisting of IgG isotype controls are displayed in Supplementary Figure 7.

Images Analysis
Digital image analyses (DIA) were undertaken to quantify the probe signal in miR-26b and SMAD1-stained human aortic tissues. All the imaging analyses were done as blinded analyses to ensure the objectivity of the results. Briefly, all tissues were normalised to the same brightfield parameters using the ZEN 2 Blue edition (ZEISSZEN Microscopy Software; RRID:SCR_013672). Five ROIs (1000 x 1000 μm) were selected across the tissue per individual sample to be processed for image analysis using FIJI Image J[5]. Using the Colour Deconvolution Plugin, images were separated into 3 channels comprised of nuclei, probe and background signal[6]. The nuclei-stained channels were thresholded using the Otsu threshold. The nuclei were measured using the "Analyse particles" function in FIJI. The Trainable Weka Segmentation Plugin was used to quantify the target signal. Trainable Weka Segmentation allows for pixel-based segmentation by implementing machine-learning algorithms on a set of features from an image[7]. Image segmentation consisted of three classes of nuclei, probe and background signal. Upon successful segmentation, the resulting images were thresholded using the Otsu threshold to exclude background and nuclei. Similarly, the resulting particles were measured using the "Analyse particles" function as previously done. For the miR-26b quantification output, miR-26b probe counts/nuclei counts were measured, while for SMAD1, the percentage of positive-stained cells was utilised.

Bulk RNA-sequencing and bioinformatics analysis
Bulk RNA-seq to determine the transcriptomes of the aorta of 6-month-old age male mice (wildtype $n = 3$ and miR26bKO $n = 3$) was performed by Beijing Genomics Institute (BGI) Company (Shenzhen, China). The sequencing was performed on a DNBSEQ system. Bioinformatics analysis was conducted with the online platform Dr. Tom (BGI Company). Only genes with transcripts per million (TPM) > 1 were analysed. Differentially expressed genes (DEGs) were identified using the DEGseq2 method and screened with the Padj value ≤ 0.05 and log2FC ≥ 1 criteria.

Network analysis and miRNA target predictions
Cytoscape (Version 3.9.1) is a freely available software tool which facilitates the creation of networks, allowing for the visualisation of molecular interactions (https://cytoscape.org/) [8]. Additional features are present in Cytoscape, in the form of Apps. StringApp (Version 2.0.3) was utilised to import established gene to gene interactions through the protein query. DIANA-miRPath v4.0[9] and TargetScan 8.0[10] was utilised for miR-26b target gene prediction and enrichment.

Single-cell RNA-sequencing

Single-cell preparation: The single-cell suspension of aortic cells was performed based on a published enzymatic digestion protocol[11]. Briefly, isolated whole aortas were finely cut and incubated in enzyme digestion buffer (400 U/mL collagenase type I, 120 U/mL collagenase type XI, 60 U/mL hyaluronidase and 20mM HEPEs in DPBS containing calcium) for 50 minutes at 37°C. The cell suspension was strained through a 100µM filter and washed with FACS buffer for 10 minutes at 4°C. After centrifugation at 300 *g*, the cells were resuspended in 1mL FACS buffer for FACS sorting. Cell suspensions were washed and re-suspended in PBS at 4 °C and analysed using a BD 5 L LSR Fortessa and BD FACSDiva software (BD Biosciences). To exclude dead cells, DAPI was added before flow cytometry analysis. Cells were sorted using a FACS Aria II instrument and BD FACSDiva software (BD Biosciences).

Library preparation and sequencing: For single-cell RNA-seq experiments, cells were prepared using 10X Genomics Chromium Next GEM Single Cell 3' Reagent Kits v3.1 with Feature Barcode technology for cell multiplexing (CG000389) following manufacturer instructions. After resuspension and before incubation with antibodies for FACS sorting, cells were stained using 10X Genomics CellPlex CMO oligos to hash individual samples (Supplementary Table S3). Three aortas were pooled per sample, and after FACS sorting, n=3 samples per group (WT vs KO) were analysed. Each pool was resuspended in an appropriate volume of PBS+1%BSA, and a maximum of 60000 cells per pool were loaded on a 10X Genomics Chip G with V3.1 gel beads and partitioning oil. Cells were then encapsulated with gel beads using a 10X Genomics Chromium X instrument. After encapsulation and cDNA synthesis, each reaction was amplified by PCR, and both low- and high-MW fractions were separately purified. The high-MW fraction containing cDNA was fragmented, ligated with sequencing adapters and amplified to generate gene expression (GEX) libraries. The low-MW fraction was further amplified with sequencing adapters to generate multiplexing (MP) libraries. All libraries were pooled at a molar ratio of GEX:MP=10:1 and sequenced on an Illumina NextSeq 2000 P3 flow cell with 100 cycles configuration.

Single-cell RNA data analysis: The single-cell RNA-Seq data were analysed using the cellranger multi command from 10X Genomics Cell Ranger v7.1.0 with a MAC value of 0.5 to provide demultiplexed cell-specific gene expression data. As an alternative approach, the nf-core scRNA-Seq pipeline (10.5281/zenodo.3568187) v2.3.0 was also run on the data using both Salmon v1.10.0 paired with simpleaf v0.10.0, and cellranger count from 10X Genomics Cell Ranger v7.1.0._The single-cell RNA-sequencing dataset was then processed, explored, and visualised using Trailmaker (Parse Biosciences): https://app.trailmaker.parsebiosciences.com/. The Data Processing settings are in Supplementary Table S4. Analyse cell-cell communication networks was performed in ICARUS (Interactive single Cell RNA-seq Analysis with R shiny Using Seurat) [12] webserver using the CellChat R package[13].

SMAD1 3'UTR Luciferase assay

Luciferase assay has been performed as previously described[14]. SMAD1 3′UTR vector was purchased from SwitchGear Genomics (The LightSwitch™ 3′UTR Reporter GoClone® Collection). The miR-26b binding sites were mutated using The GeneTailor™ Site-Directed Mutagenesis System (ThermoFisher Scientific).

Primers for 3′ UTR mutation are as follows: SMAD1 mut #1, forward 5′-ACTATTGAGCCTTGCATGTgtggatAGGATGGATGA-3′ and reverse 5′-TCATCCATCCTatccacACATGCAAGGCTCAATAGT -3′; SMAD1 mut #2 forward 5′ -

ACAAAGGAGCCTTGATAATgtggatCCTCTGTGAC-3′ and reverse 5′ - GTCACAGAGGatccacATTATCAAGGCTCCTTTGT -3’. Luciferase constructs were transfected into HEK293T cells with miR-26b mimics or p-SV-β-gal control vector. Cells were cultured for 48 hr and assayed with the Luciferase and β-Galactosidase Reporter Assay Systems (Promega). Luciferase values were normalised to protein concentration and β-galactosidase activity.

Pressure myography

The structural and mechanical properties of MRAs were studied with a pressure myograph (Danish Myo Tech, model P100, J.P Trading I/S, Aarhus, Denmark) as described in [15]. Briefly, the vessel was placed on two glass micro-cannulae, secured with surgical nylon sutures, and the vessel length was adjusted so that the vessel walls were parallel without stretch. Intraluminal pressure was then raised to 140 mmHg, and the artery was unbuckled by adjusting the cannulae. The segment was then set to a pressure of 70 mmHg and allowed to equilibrate for 60 min at 37°C in calcium-free KHS ($0Ca^2$+; omitting calcium and adding 10 mM EGTA) gassed with a mixture of 95% O2 and 5% CO_2. Intraluminal pressure was reduced to 3mmHg. A pressure–diameter curve was obtained by increasing intraluminal pressure in 20 mmHg steps between 20 and 140 mmHg. Internal and external diameters (D_{i0Ca} and D_{e0Ca}) were measured for 5 minutes at each intraluminal pressure. Mechanical properties were represented as stress-strain curves.

Atomic force microscopy

QI-Imaging was carried out on a JPK NanoWizard 4XP (Bruker, Germany) mounted on an Axio Observer 5 inverted microscope (Zeiss, Germany) using a CP-qp-CONT-SiO-C-5 probe (sQube), with a nominal spring constant of 0.1 N/m and a 6.62 µm Spherical tip. Reference force curves were taken on a clean glass slide before and after experiments to determine the cantilevers sensitivity and thermal tuning was done to obtain the spring constant. Tissue samples were thawed and rinsed in PBS to remove the embedding matrix, and then they were kept in PBS for the duration of the experiment. Each sample was optically imaged using DirectOverlay to place the cantilever accurately. For QI-imaging 50x50 µm regions were imaged at 100x100 pixels with a setpoint of 10 nN; a Z-length of 2 µm; and a speed of 20 µm/s to obtain the mechanical properties.
Analysis: QI images were analysed using the Hertz model modified for a spherical indenter with JPK Data Processing software (Bruker, Germany). Images were further processed using Gwyddion 2.61 Software (Czech Metrology Institute, Czech Republic).

Statistical Analysis
Normal data distribution was assessed using Kolmogorov-Smirnov or Shapiro-Wilk's tests as appropriate. Continuous variables are shown as mean ± standard error of the mean (SEM). Categorical variables are presented as counts (percentages). Normally distributed variables were analysed using the unpaired two-tailed Student's t-test (two groups comparisons) or one-way analysis of variance (multiple groups comparisons ANOVA), as appropriate. ANOVA post-hoc analysis was performed using the Bonferroni approach as appropriate. Data with two factors were analysed by two-way ANOVA followed by Tukey post hoc analysis. Non-parametric tests, including the Wilcoxon/Mann–Whitney U test or the Kruskal-Wallis test, were used for data that was not normally distributed. Pearson or Spearman linear regression was calculated to study the relationship among continuous variables of interest. A p-value < 0.05 was considered statistically significant. Analyses were performed using GraphPad Prism v5.0.

Data availability
RNA-sequencing data generated during this study are available in the Gene Expression Omnibus repository (http://www.ncbi.nlm.nih.gov/geo/) and accessible through the Gene Expression Omnibus series accession numbers GSE281028 (scRNA-seq) and GSE281279 (bulk RNA-seq).

SUPPLEMENTARY FIGURES

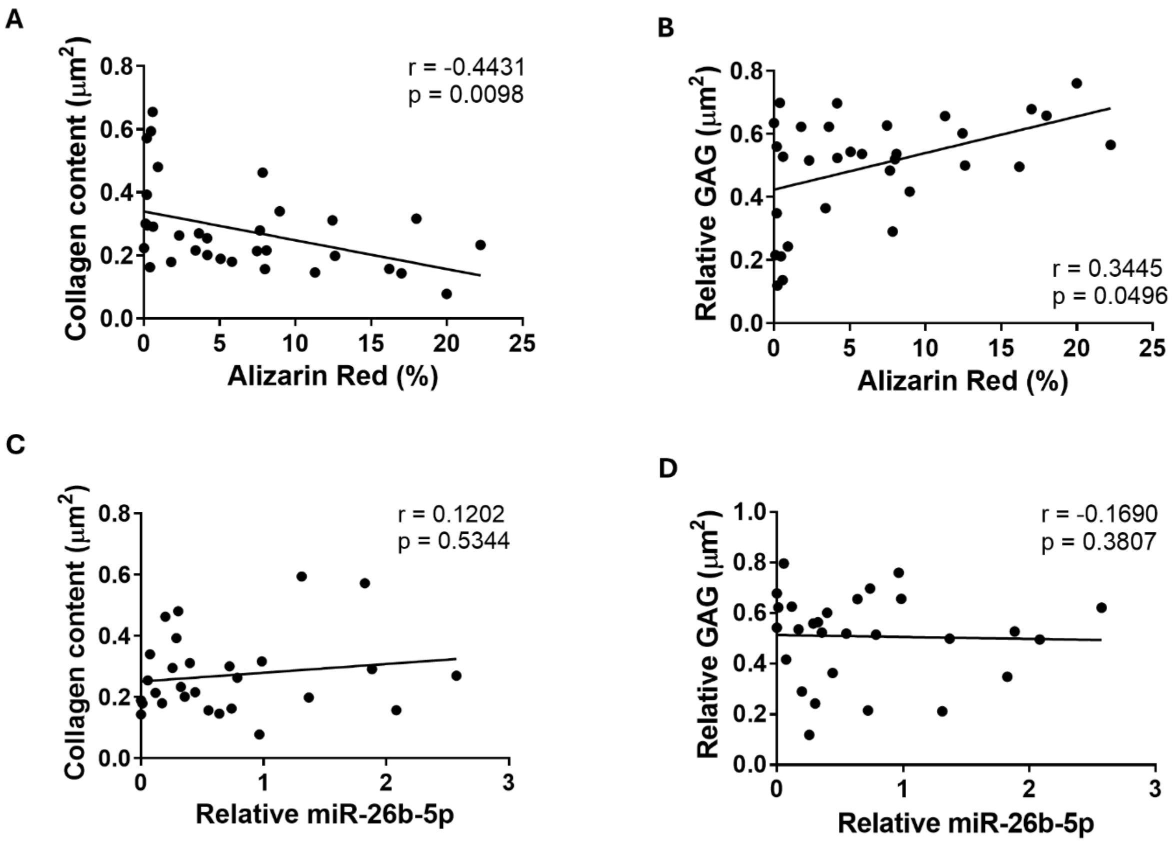

Supp. Figure 1: Correlation between miR-26b and collagen or glycosaminoglycan (GAG) accumulation in human aortic samples.

(**A**) Correlation of collagen content and calcium levels (r=-0.4431, p=0.0098, Spearman correlation) and (**B**) correlation of GAG accumulation and calcium levels (r=0.3445, p=0.0496, Spearman correlation). No significant correlation was found between the level of aortic miR-26b-5p expression and (**C**) collagen content (r=0.1202, p=0.5344) or (**D**) GAG accumulation (r=-0.1690, p=0.3807). N=35: n=19 low (5 female/14 male) and n=16 medium/high (4 female/12 male).

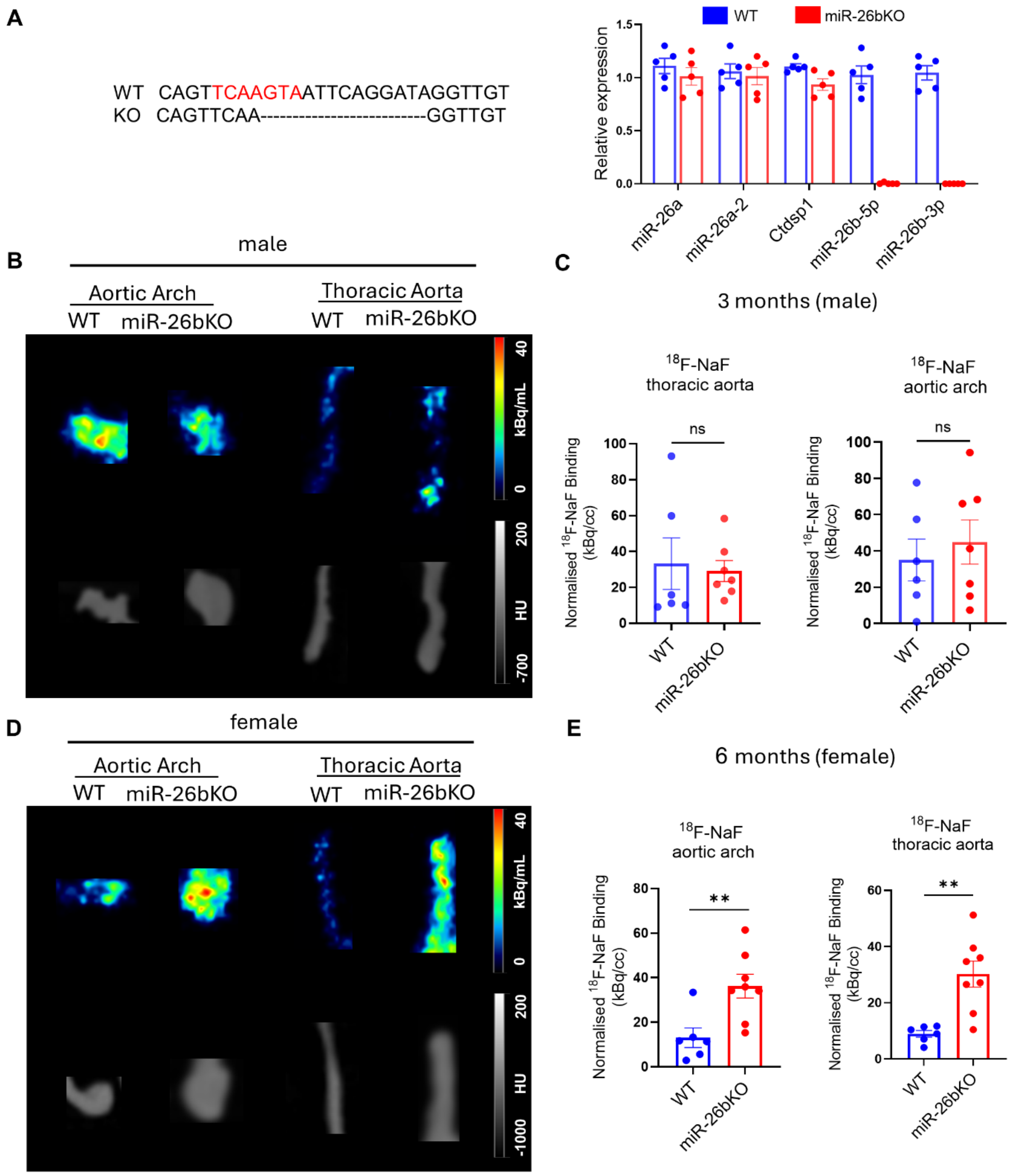

Supp. Figure 2: Microcalcification analysis in male mice at 3 months of age and female mice at 6 months of age (WT vs miR-26bKO).

(**A**) DNA sequencing analysis demonstrated deletions (13bp deletion) in the miR-26b sequences with the partial loss of the miR-26b seed sequence (red letters) in the KO mice (left panel). miR-26b-5p and miR-26b-3p expression was completely abolished in 8-week-old miR-26bKO aortas (right panel). Representative ex-vivo micro-PET/CT scans of the heart and descending aorta in (**B**) 3-month-old male WT and miR-26bKO mice. The colours correspond to the intensity of the ^{18}F-NaF (hydroxyapatite marker) signal and (**C**) quantification of microcalcification in the aortic arch and thoracic aorta. Representative ex-vivo micro-PET/CT scans of the heart and descending aorta in (**D**) 6-month-old female WT and miR-26bKO mice and (**E**) quantification of microcalcification in the aortic arch and thoracic aorta. For C: ns=non-significant; Student's unpaired t-test, n=6-7/group. For E: $p<0.01$ vs WT; Student's unpaired t-test, n=6-8/group. All data are mean ± SEM.

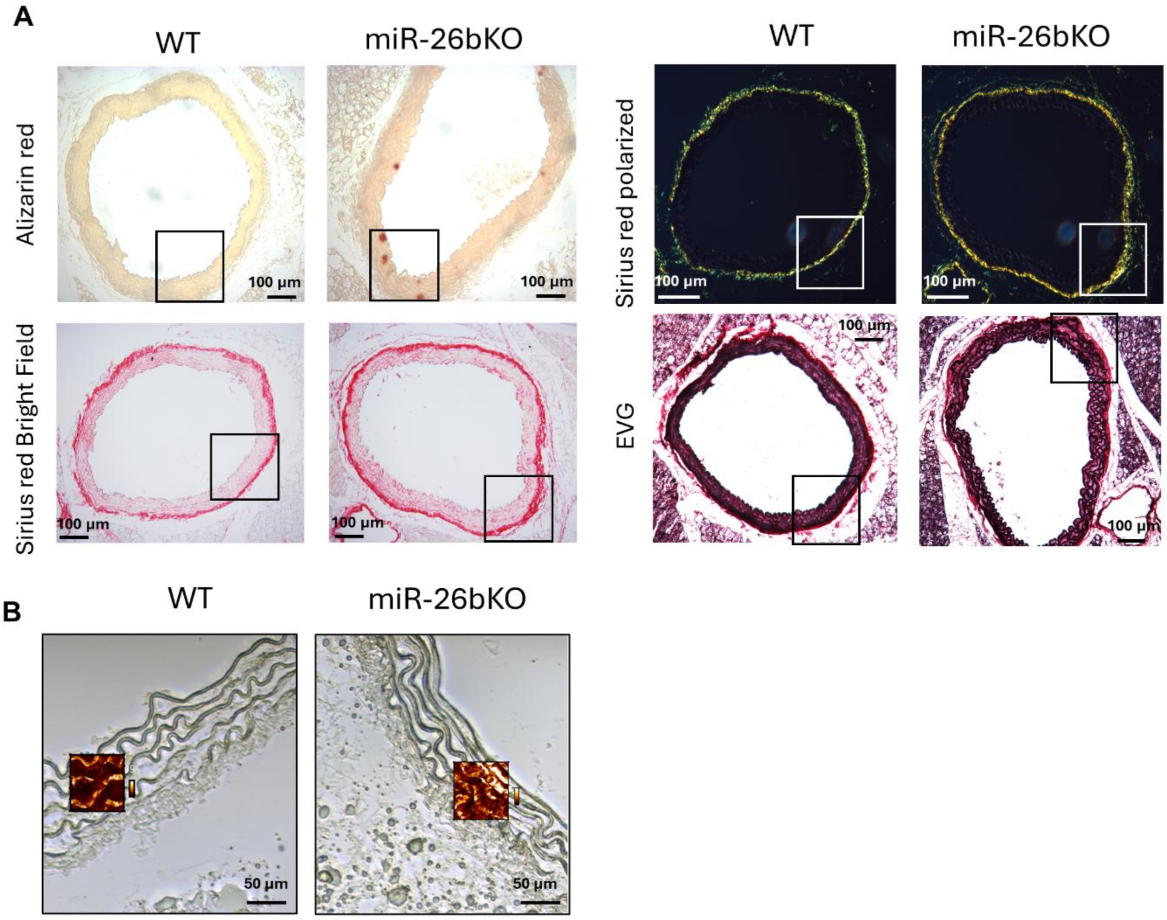

Supp. Figure 3: Low-magnification images of the aortic rings.
(**A**) Histological analysis of WT and miR-26bKO aortas using Alizarin Red, Sirius Red and Elastic Van Giesson (EVG). (**B**) Atomic force microscopy representative images.

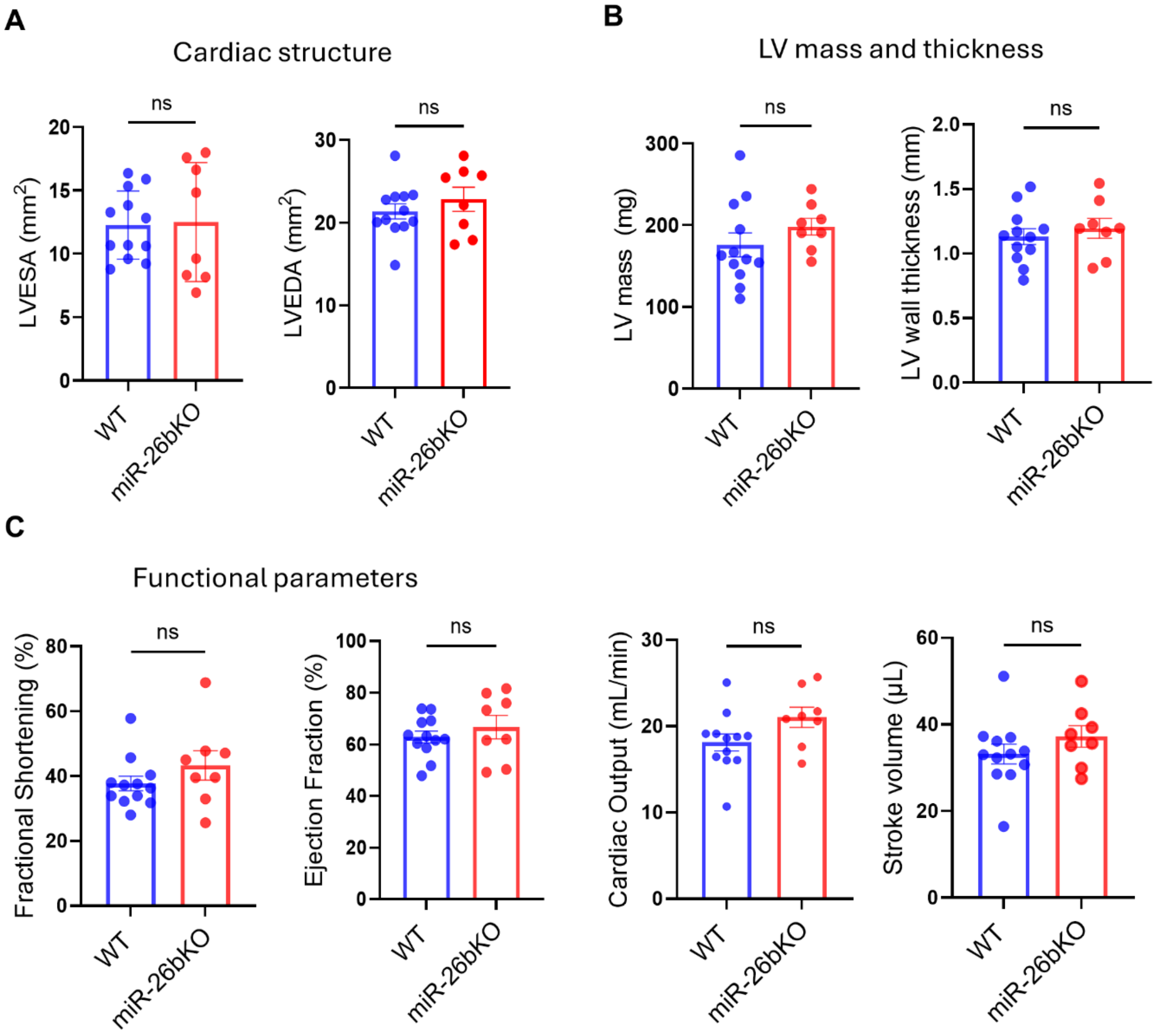

Supp. Figure 4: Echocardiography revealed that cardiac function and structure were unaltered in miR-26b KO mice at 6 months of age compared to wild-type mice.
(**A**) Analysis measurements of WT and miR-26b consisting of left ventricular end-systolic area (LVESA; mm^2) and left ventricular end-diastolic area (LVEDA; mm^2), (**B**) left ventricular (LV) mass (mg) and wall thickness measurements (mm), (**C**) fractional shortening (%), ejection fraction (%), cardiac output (mL/min) and stroke volume (µL). Male mice have been used in these experiments. Student's unpaired t-test. ns = non-significant. n=8-12 per group. All data are mean ± SEM.

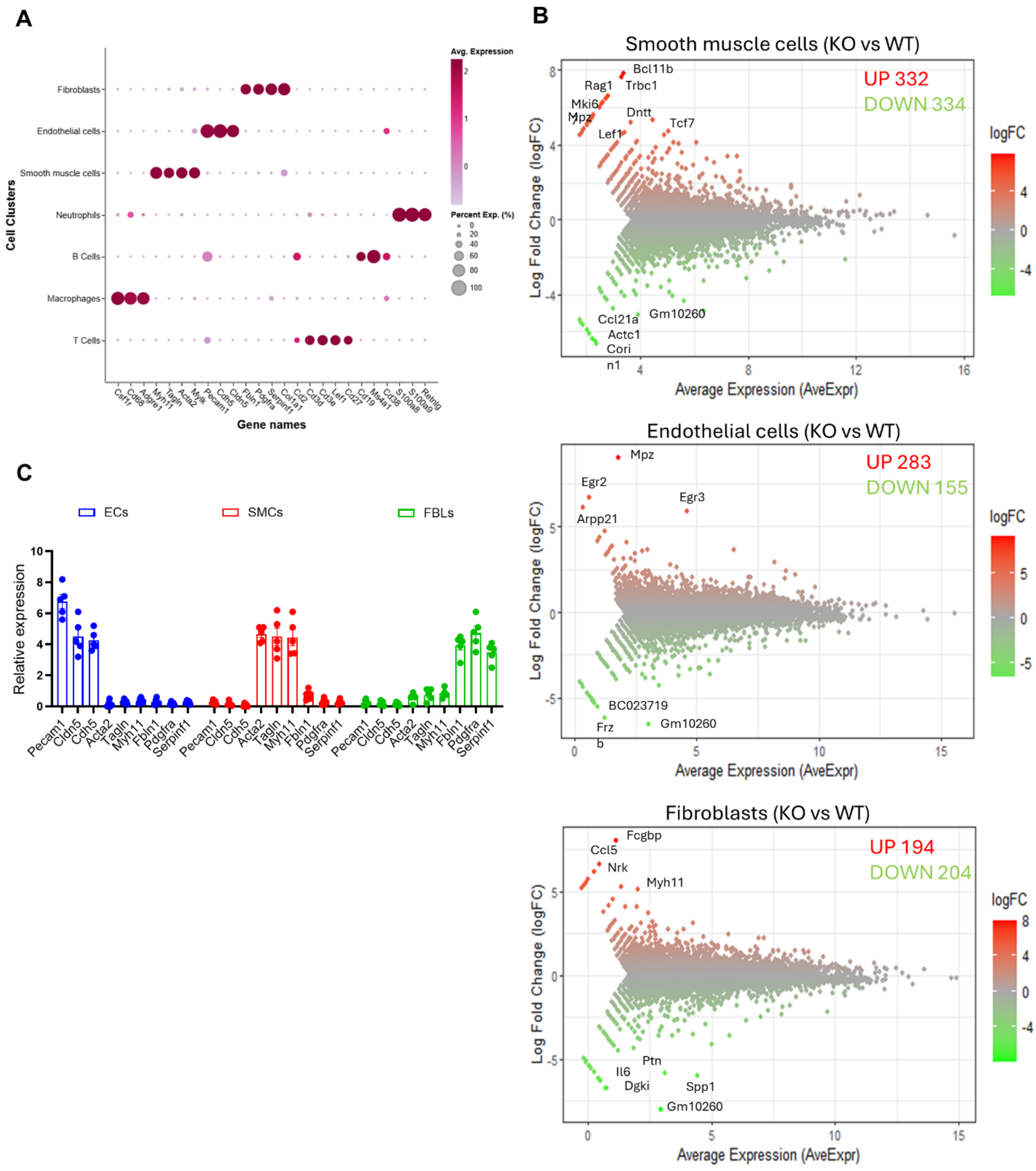

Supp. Figure 5: Cluster frequency analysis and characterisation of aortic cells.
(**A**) Dot plot representing the gene markers for the cell types utilised for manual cluster annotation. Average expression is represented by colour, with increased expression represented by a darker colour versus decreased expression with a lighter colour. Percentage (%) expression is displayed according to the size of the dot, with a larger dot representing a higher percentage of a cell expressing a gene. (**B**) MA plots to analyse and interpret differentially expressed genes (DEGs) (log2 fold change = 1.5; adjusted p-value≤0.05). Genes with significantly increased expression are in red dots, while those with significantly decreased expression are marked with green dots. This comparison is made between the endothelial cells (ECs), smooth muscle cells (SMCs), and fibroblasts (FBLs) in miR-26bKO mice *vs.* WT mice. (**C**) Expression of ECs, SMCs and FBLs marker genes in cells isolated or sorted from WT aortas. All data are mean ± SEM (n=5 /group).

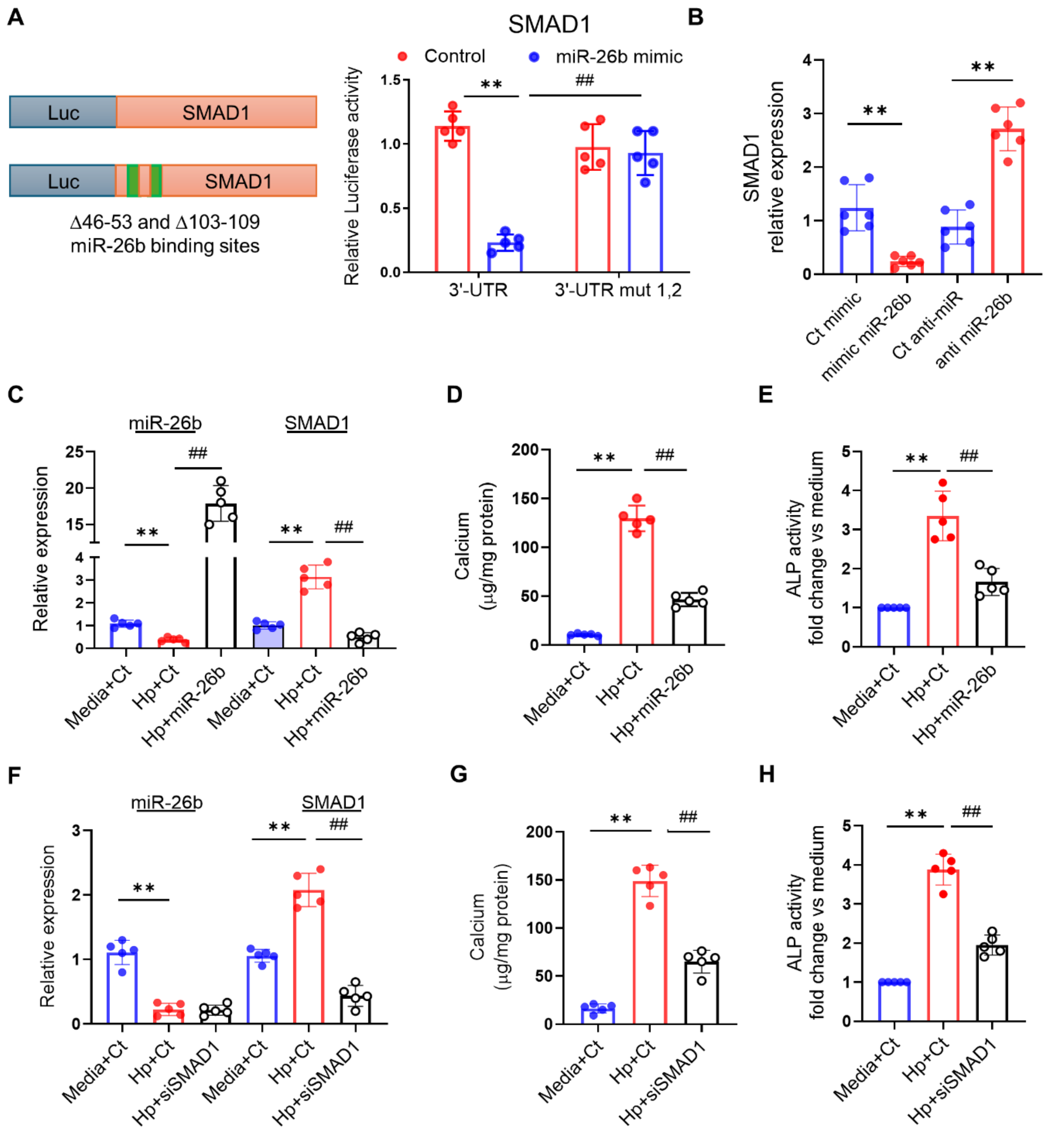

Supp. Figure 6: Validation of SMAD1 as miR-26b target gene.
(**A**) Diagram of 3'UTR SMAD1 deleted sequence and SMAD1 luciferase assay on WT and mutated SMAD1 3'UTR (**p<0.01 vs. Control. ##p<0.01 vs miR-26b mimic. One-way ANOVA. n=5 per group). (**B**) SMAD1 expression after miR-26b mimic and anti-miR-26b transfection (**p<0.01 vs. Control. Student's unpaired t-test. n=6 per group). (**C**) Expression of miR-26b-5p and SMAD1 in human aortic smooth muscle cells (HAoSMCs) cultured in Hp (2.6 mM) or control medium+/- miR-26b mimic transfection. (**D**) Calcium deposition and (**E**) ALP activity of HAoSMCs transfected with miR-26b mimic in Hp media for 10 days. (**F**) Expression of miR-26b-5p and SMAD1 in HAoSMCs cultured in Hp or control media+/- miR-26b mimic transfection. (**G**) Calcium deposition and (**H**) ALP activity of HAoSMCs transfected with miR-26b mimic in Hp media for 10 days. **p<0.01 *vs* Media+Ct; ##p<0.01 *vs* CaP+Ct. Two-way ANOVA. n = 5 per group. All data are mean ± SEM.

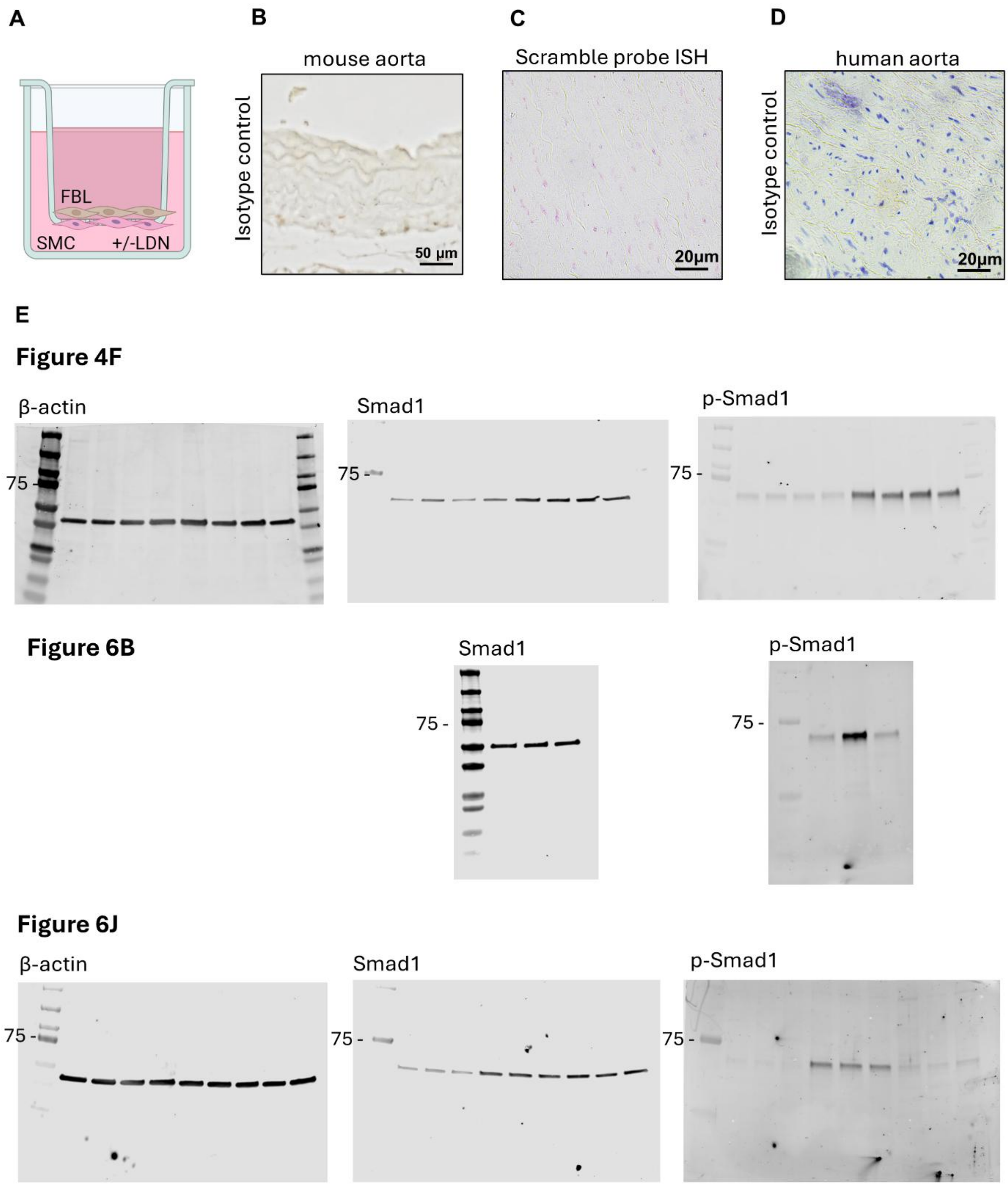

Suppl. Figure 7: (**A**) Smooth muscle cells/Fibroblasts co-culture system on Transwell™. (**B**) Isotype control for SMAD1 antibody in mouse aortas. (**C**) The negative control for ISH is the scrambled probe with the same sequence as the miR-26b probe, but the nucleotides are placed randomly. (**D**) Isotype control for SMAD1 antibody in human aortas. **(E)** Uncropped Western blots in Figure 4 and Figure 6.

Table S1: Clinical data with concomitant aortopathy and aortic valve disease

Variable	Stats	Total	Low Ca^{+2}	Medium/High Ca^{+2}	p-value
n (female/male)		35	19 (5f/14m)	16 (4f/12m)	1.0000
Age	Mean ± SD	63.88±10.25	64.32±10.50	63.33±10.26	0.9250
Weight (Kg)	Mean ± SD	77.58±14.78	77.45±17.81	77.73±10.69	0.9559
Height (cm)	Mean ± SD	170.40±8.70	171.4±9.05	169.1±8.38	0.4450
Body mass index (Kg/m^2)	Mean ± SD	26.60±4.02	26.09±4.47	27.20±3.45	0.4260
DM	n (%)	7 (20.0)	2 (10.53)	5 (31.250)	1.0000
Renal insufficiency	n (%)	1 (4.54)	1 (12.5)	0	0.4444
HTA	n (%)	17 (48.57)	12 (63.16)	5 (31.250)	0.0922
HLP	n (%)	17 (48.57)	8 (42.10)	9 (56.25)	0.5051
CAD	n (%)	6 (18.18)	3 (15.78)	3 (21.43)	1.0000
**Type of AVD**					
Aortic stenosis	n (%)	9 (28.12)	3 (17.65)	6 (40.00)	0.2433
Aortic regurgitation	n (%)	18 (56.25)	10 (58.82)	8 (53.33)	1.0000
Double aortic valve lesion	n (%)	4 (12.50)	4 (23.53)	0	0.1041
None	n (%)	1 (3.12)	0	1 (6.25)	0.4688
**Drug medicines**					
ACEI/ARB	n (%)	18 (51.43)	11 (57.89)	7 (43.75)	0.5051
Diuretics	n (%)	11 (31.43)	6 (31.58)	5 (31.25)	1.0000
β-blockers	n (%)	15 (42.86)	8 (42.10)	7 (43.75)	1.0000
Statins	n (%)	18 (51.43)	9 (0.47)	9 (56.25)	0.7380
**Biochemical analyses**					
TGCs (mg/dL)	Mean ± SD	97.04±50.66	86.71±44.40	111.5±57.56	0.1259
Total cholesterol (mg/dL)	Mean ± SD	180.10±43.25	186.1±45.77	172.40.39	0.3307
HDL (mg/dL)	Mean ± SD	50.26±11.82	50.76±14.29	49.64±8.40	0.7976
LDL (mg/dL)	Mean ± SD	107.30±39.24	111.1±41.20	102.80±37.72	0.5679
**Echocardiographic parameters**					
EF %	Mean ± SD	59.60±10.12	61.00±8.48	58.00±11.85	0.4276
**Histological DIA**					
Calcium content (A.U)	Mean ± SEM	5.19±1.08	0.70±0.18	10.53±1.50	<0.0001
Fibrosis content (A.U)	Mean ± SEM	0.28±0.03	0.35±0.05	0.21±0.02	0.0045
GAGs content (A.U)	Mean ± SEM	0.51±0.03	0.40±0.06	0.58±0.03	0.0092

Table S2: genotyping primers and gRNA sequences

	**miR-26b gRNA sequence**
gRNA_1	ATCCATTAATACGACTCACTATA**GGGACCCAGTTCAAGTAATTC**GTTTTAGAGCTAGAAATAGCAAGTTAAAATAAGGCTAGTCCGTTATCAACTTGAAAAAGTGGCACCGAGTCGGTGCTTTTTT
gRNA_2	ATCCATTAATACGACTCACTATA**GGGCCGGTGCCCTGCAGCCT**GTTTTAGAGCTAGAAATAGCAAGTTAAAATAAGGCTAGTCCGTTATCAACTTGAAAAAGTGGCACCGAGTCGGTGCTTTTTT
	**miR-26b Genotyping**
	F: ACTCCAGAGATGGAGCCAGA
	R: CAAATGCAGGAGTCCAGGTT

Table S3: 10X Genomics CellPlex Cell Multiplexing Oligos (CMO)

Sample	id	sequence
WT1	CMO307	AAGCTCGTTGGAAGA
WT2	CMO308	CGGATTCCACATCAT
WT3	CMO309	GTTGATCTATAACAG
KO1	CMO310	GCAGGAGGTATCAAT
KO2	CMO311	GAATCGTGATTCTTC
KO3	CMO312	ACATGGTCAACGCTG

Table S4: scRNA-seq Data Processing Settings

Data processing settings	
[1-classifier.WT]	FDR = 0.01
[1-classifier.KO]	FDR = 0.01
[2-cellSizeDistribution.WT]	binStep = 200<br>minCellSize = 478
[2-cellSizeDistribution.KO]	binStep = 200<br>minCellSize = 757
[3-mitochondrialContent.WT]	method = absoluteThreshold<br>binStep = 0.3<br>maxFraction = 0.08138598
[3-mitochondrialContent.KO]	method = absoluteThreshold<br>binStep = 0.3<br>maxFraction = 0.06230307
[4-numGenesVsNumUmis.WT]	regressionType = linear<br>p.level = 0.0005959476
[4-numGenesVsNumUmis.KO]	regressionType = linear<br>p.level = 0.0007955449
[5-doubletScores.WT]	binStep = 0.02<br>probabilityThreshold = 0.6687695
[5-doubletScores.KO]	binStep = 0.02<br>probabilityThreshold = 0.8573509
[6-dataIntegration.awsConfig]	region = eu-west-1
[6-dataIntegration.dataIntegration]	method = harmony<br>numGenes = 2000<br>normalisation = logNormalize
[6-dataIntegration.dimensionalityReduction]	method = rpca<br>numPCs = 28
[7-configureEmbedding.awsConfig]	region = eu-west-1
[7-configureEmbedding.embeddingSettings]	method = umap<br>distanceMetric = cosine<br>minimumDistance = 0.9
[7-configureEmbedding.clusteringSettings]	method = louvain<br>resolution = 0.2

REFERENCES

1. Fletcher AJ, Nash J, Syed MBJ, Macaskill MG, Tavares AAS, Walker N, Salcudean H, Leipsic JA, Lim KHH, Madine J, Wallace W, Field M, Newby DE, Bouchareb R, Seidman MA, Akhtar R, Sellers SL. Microcalcification and Thoracic Aortopathy: A Window Into Disease Severity. *Arterioscler Thromb Vasc Biol* 2022;**42**:1048-1059.
2. Upreti A, Hoang TV, Li M, Tangeman JA, Dierker DS, Wagner BD, Tsonis PA, Liang C, Lachke SA, Robinson ML. miR-26 Deficiency Causes Alterations in Lens Transcriptome and Results in Adult-Onset Cataract. *Invest Ophthalmol Vis Sci* 2024;**65**:42.
3. Boergermann JH, Kopf J, Yu PB, Knaus P. Dorsomorphin and LDN-193189 inhibit BMP-mediated Smad, p38 and Akt signalling in C2C12 cells. *Int J Biochem Cell Biol* 2010;**42**:1802-1807.
4. Schmittgen TD, Livak KJ. Analyzing real-time PCR data by the comparative C(T) method. *Nat Protoc* 2008;**3**:1101-1108.
5. Schindelin J, Arganda-Carreras I, Frise E, Kaynig V, Longair M, Pietzsch T, Preibisch S, Rueden C, Saalfeld S, Schmid B, Tinevez JY, White DJ, Hartenstein V, Eliceiri K, Tomancak P, Cardona A. Fiji: an open-source platform for biological-image analysis. *Nat Methods* 2012;**9**:676-682.
6. Landini G, Martinelli G, Piccinini F. Colour deconvolution: stain unmixing in histological imaging. *Bioinformatics* 2021;**37**:1485-1487.
7. Arganda-Carreras I, Kaynig V, Rueden C, Eliceiri KW, Schindelin J, Cardona A, Sebastian Seung H. Trainable Weka Segmentation: a machine learning tool for microscopy pixel classification. *Bioinformatics* 2017;**33**:2424-2426.
8. Shannon P, Markiel A, Ozier O, Baliga NS, Wang JT, Ramage D, Amin N, Schwikowski B, Ideker T. Cytoscape: a software environment for integrated models of biomolecular interaction networks. *Genome Res* 2003;**13**:2498-2504.
9. Tastsoglou S, Skoufos G, Miliotis M, Karagkouni D, Koutsoukos I, Karavangeli A, Kardaras FS, Hatzigeorgiou AG. DIANA-miRPath v4.0: expanding target-based miRNA functional analysis in cell-type and tissue contexts. *Nucleic Acids Res* 2023;**51**:W154-W159.
10. McGeary SE, Lin KS, Shi CY, Pham TM, Bisaria N, Kelley GM, Bartel DP. The biochemical basis of microRNA targeting efficacy. *Science* 2019;**366**.
11. Hu D, Yin C, Mohanta SK, Weber C, Habenicht AJ. Preparation of Single Cell Suspensions from Mouse Aorta. *Bio Protoc* 2016;**6**.
12. Jiang A, Lehnert K, You L, Snell RG. ICARUS, an interactive web server for single cell RNA-seq analysis. *Nucleic Acids Res* 2022;**50**:W427-W433.
13. Jin S, Plikus MV, Nie Q. CellChat for systematic analysis of cell-cell communication from single-cell transcriptomics. *Nat Protoc* 2024.
14. Miscianinov V, Martello A, Rose L, Parish E, Cathcart B, Mitic T, Gray GA, Meloni M, Al Haj Zen A, Caporali A. MicroRNA-148b Targets the TGF-beta Pathway to Regulate Angiogenesis and Endothelial-to-Mesenchymal Transition during Skin Wound Healing. *Mol Ther* 2018;**26**:1996-2007.
15. Briones AM, Xavier FE, Arribas SM, Gonzalez MC, Rossoni LV, Alonso MJ, Salaices M. Alterations in structure and mechanics of resistance arteries from ouabain-induced hypertensive rats. *Am J Physiol Heart Circ Physiol* 2006;**291**:H193-201.
